# Modulation of the 20S Proteasome Activity by Porphyrin Derivatives Is Steered through Their Charge Distribution

**DOI:** 10.3390/biom12060741

**Published:** 2022-05-24

**Authors:** Marco Persico, Anna Maria Santoro, Alessandro D’Urso, Danilo Milardi, Roberto Purrello, Alessandra Cunsolo, Marina Gobbo, Roberto Fattorusso, Donatella Diana, Manuela Stefanelli, Grazia R. Tundo, Diego Sbardella, Massimo Coletta, Caterina Fattorusso

**Affiliations:** 1 Department of Pharmacy, University of Naples “Federico II”, Via D. Montesano 49, 80131 Napoli, Italy; m.persico@unina.it; 2 National Research Council, Institute of Crystallography, Sede Secondaria di Catania, Via Paolo Gaifami 18, 95126 Catania, Italy; danilo.milardi@cnr.it; 3 Department of Chemical Sciences, University of Catania, Viale A. Doria 6, 95125 Catania, Italy; adurso@unict.it (A.D.); rpurrello@unict.it (R.P.); alessandracunsolo86@gmail.com (A.C.); 4 Department of Chemical Sciences, University of Padua, Via F. Marzolo, 1, 35131 Padova, Italy; marina.gobbo@unipd.it; 5 Department of Environmental, Biological and Pharmaceutical Sciences and Technologies, University of Campania “Luigi Vanvitelli”, Via Vivaldi 43, 81100 Caserta, Italy; roberto.fattorusso@unicampania.it; 6 National Research Council, Insitute of Biostructures and Bioimaging, Via Mezzocannone 16, 80134 Napoli, Italy; donatella.diana@cnr.it; 7 Department of Chemical Sciences and Technologies, University of Roma Tor Vergata, Via della Ricerca Scientifica, 00133 Roma, Italy; manuela.stefanelli@uniroma2.it; 8 Department of Clinical Sciences and Translational Medicine, University of Roma Tor Vergata, Via Montpellier 1, 00133 Roma, Italy; grazia.raffaella.tundo@uniroma2.it; 9 IRCCS-Fondazione BIETTI, Rome, Italy; diego.sbardella@fondazionebietti.it

**Keywords:** h20S proteasome modulators, cationic porphyrins, NMR, kinetic studies, molecular docking

## Abstract

Cationic porphyrins exhibit an amazing variety of binding modes and inhibition mechanisms of 20S proteasome. Depending on the spatial distribution of their electrostatic charges, they can occupy different sites on α rings of 20S proteasome by exploiting the structural code responsible for the interaction with regulatory proteins. Indeed, they can act as competitive or allosteric inhibitors by binding at the substrate gate or at the grooves between the α subunits, respectively. Moreover, the substitution of a charged moiety in the peripheral arm with a hydrophobic moiety revealed a “new” 20S functional state with higher substrate affinity and catalytic efficiency. In the present study, we expand our structure–activity relationship (SAR) analysis in order to further explore the potential of this versatile class of 20S modulators. Therefore, we have extended the study to additional macrocyclic compounds, displaying different structural features, comparing their interaction behavior on the 20S proteasome with previously investigated compounds. In particular, in order to evaluate how the introduction of a peptidic chain can affect the affinity and the interacting mechanism of porphyrins, we investigate the MTPyApi, a porphyrin derivatized with an Arg–Pro-rich antimicrobial peptide. Moreover, to unveil the role played by the porphyrin core, this was replaced with a corrole scaffold, a “contracted” version of the tetrapyrrolic ring due to the lack of a methine bridge. The analysis has been undertaken by means of integrated kinetic, Nuclear Magnetic Resonance, and computational studies. Finally, in order to assess a potential pharmacological significance of this type of investigation, a preliminary attempt has been performed to evaluate the biological effect of these molecules on MCF7 breast cancer cells in dark conditions, envisaging that porphyrins may indeed represent a powerful tool for the modulation of cellular proteostasis.

## 1. Introduction

The ubiquitin–proteasome system (UPS) is the main actor in the control of the turn-over of most cellular proteins, displaying crucial roles in several facets of cell life, such as cell cycle, apoptosis, DNA repair, antigen presentation, inflammation, cellular response to environmental stress, morphogenesis of neuronal networks, and retinal homeostasis [1,2,3,4]. This system displays a hierarchical organization, encompassing two intertwined and consecutive steps: (i) the covalent attachment of the ubiquitin chain to substrates and (ii) the degradation by the 26S proteasome of ubiquitin-tagged substrates [5,6]. The endpoint of the UPS is the 26S multisubunit proteolytic machine, composed of a catalytic core particle (CP or 20S proteasome), which houses the three proteolytic activities, capped by one or two 19S regulatory particle(s) (RPs) [7,8]. The 19S is a sophisticated assembly, structured in two modules: the base and the lid. The base interacts directly with the 20S, and it is constituted by six ATPases subunits (i.e., Rpt1–6), being involved in substrate unfolding and translocation through the gate into the 20S catalytic chamber, and three non-ATPase subunits (i.e., Rpn1, Rpn2, Rpn13), which provide multiple binding sites for ubiquitin and ubiquitin-like proteins [9,10]. The peripheral lid braces one side of the base and is composed of nine non-ATPase subunits (i.e., Rpn3, Rpn5–Rpn9, Rpn11, Rpn12, and Sem1), which are deputed to substrate recognition and de-ubiquitination. Besides 19S, 20S may bind other RPs, such as PA28 (REGα/11S) and PA200 [11,12]. PA28 is an ATP-independent activating complex of human 20S (h20S) consisting of seven protein subunits with three identified isoforms (PA28α-γ) [13]. PA200 is also an ATP-independent activator, stimulating caspase-like (C-L) proteasomal hydrolysis almost three times more than chymotryptic-like (ChT-L) and tryptic-like (T-L) activity [12]. The 20S core particle is a hollow tube-shaped assembly composed of 28 subunits arranged in four heptameric stacked rings and structured into two outer α-rings and two inner β-rings. In the two inner beta rings (i.e., β1–7 subunits) are located at the proteolytic sites (i.e., ChT-L at the β_5_ subunits, T-L) at the β_2_ subunits, and C-L at the β_1_ subunits) [7]. The outer α-rings form a nearly flat surface with shallow grooves between adjacent α- subunits (α-grooves) that interacts with RPs and proteasome interacting proteins (PIPs) [5,14,15]. In the free h20S, the N-terminal tails of the α-subunits all point inwards to the center of the ring and the neighboring tails form “the gate”, which regulates the substrate access through a 13 Å entry pore into the central cavity [16,17]. The conformation of the gate determines if the h20S is in the “latent” (“closed”) or “activated” (“open”) state [18] since the substrate insertion through this “N-terminal gate” is the rate-limiting step of the proteasome activity [17,19,20]. As a matter of fact, RP binding to the α-ring induces the N-terminal tails’ displacement and opens the gate, facilitating the substrate translocation through an allosteric mechanism not yet fully characterized.

The 20S proteasome is not simply a machine for automatic destruction of proteins; its dynamic nature and the complex network of information and signals circulating from the catalytic sites to the gate area and *vice versa* [21] bring about an autonomous ability to work also in a naked form (without RPs) in a ubiquitin- and ATP-independent way [22]. This modality is redox-regulated and represents the preferential degradation pathway of intrinsically disordered proteins (IDPs) [23]. Due to the myriad of biochemical pathways controlled by the UPS, the proteasome has emerged over the last two decades as an excellent target for the design of new therapies. In particular, it has been observed that proteasome activity and regulation are altered in tumor cells [1], rendering this system an attractive therapeutic target for cancers.

Bortezomib is a proteasome covalent inhibitor that preferentially inhibits the ChT-L activity; such an inhibition is associated with cell cycle arrest, apoptosis, and changes in cell surface adhesion markers [24]. Its FDA approval in 2003 for the treatment of multiple myeloma (and then also for the mantle lymphoma) has largely urged the proteasome search and increased the clinical interest on proteasome inhibition. Second-generation proteasome inhibitors (carfilzomib and ixazomib) have been approved to overcome bortezomib resistance [25] thanks to an improved toxicity profile [26]. Despite the success of the proteasome inhibitors currently approved, their therapeutic use is still limited by both intrinsic and acquired resistance phenomena [25]. In the last decade, a plethora of structurally different inhibitors, either natural products or synthetic compounds, have been discovered to be effective against constitutive proteasome and/or against immunoproteasome. By contrast, proteasome activators are also potentially applicable to the reduction in proteotoxic stresses in neurodegeneration, retinal disorders, and aging, where a dysfunction of UPS is also largely reported [2,4]. As a matter of fact, there is a growing interest in finding small molecules able to allosterically regulate h20S rather than competitively inhibit it; this strategy should allow to bypass drug resistance phenomena, which, along with the onset of adverse events, represents the main drawback that limits the proteasome inhibitors’ therapeutic potentiality.

Porphyrins represent promising platforms to be engineered as competitive or allosteric proteasome regulators [27,28,29,30]. In the last few decades, these ring-shaped molecules have attracted major attention as anticancer drugs for their uses in photodynamic therapy (PDT) because of their ability to generate reactive oxygen species (ROS) upon light irradiation. Moreover, PDT, which was introduced to ophthalmology in 2000, is employed to treat vascular issues in the retina and choroid, such as corneal neovascularization and age-related macular degeneration [31,32,33]. Further, some recent investigations have evidenced how their light-independent antitumor effects may pave the way to new and promising applications. Within this context, the capacity of porphyrins to modulate the proteasome function through a remarkable variety of binding modes and inhibition mechanisms is noteworthy. They may bind to the 20S gates, inducing their partial occlusion (by competitively hindering the entrance of the substrate into the catalytic chamber) [28], or, alternatively, they may interact with different α-grooves, thus affecting the dynamic equilibrium between the open and closed state of the proteasome gates [29]. Additional binding modes, all resulting in allosteric inhibition mechanisms, involve interactions with the grooves connecting α- and β-rings, as well as with the β5 catalytic subunits on their own. This evidence suggests that the charged peripheral substituents of porphyrins interfere with the electrostatic code, represented by the charged residues in the surface of α-subunits, which is also exploited by RP to modulate the gating phenomena.

Recently, we also reported a peculiar behavior of a tri-cationic porphyrin (i.e., Tris-T4) that revealed the existence of two functional states of h20S (never described before). Furthermore, Tris-T4 cooperatively interacts with constitutive h20S proteasome, shifting the conformational equilibrium toward the functional state with an enhanced catalytic activity, behaving then as a proteasome activator [30]. Here, using a combination of enzyme assays, Nuclear Magnetic Resonance (NMR), and in silico studies, we further extended our investigation considering: (i) MTPyApi, a porphyrin derivatized with the antimicrobial peptide apidaecin, previously used in photo-inactivation studies against bacteria [34], which shares a Pro-Arg-rich motif with peptides reported to bind the h20S proteasome [35]; and (ii) TMPC, where the tetrapyrrolic porphyrin ring is replaced with a corrole core [36]. Although much less investigated than their “expanded” counterparts, corroles have exhibited very attractive anticancer properties [37]. Furthermore, we characterized the biological effect of TMPC together with those of the porphyrin-based inhibitors H_2_T4 (competitive) and pTMPyPP4 (allosteric) directly on the MCF7 breast cancer cells, trying to determine the relationship between the activity on isolated proteasome inhibition and cytotoxic effects of porphyrins in dark conditions.

Finally, we have compared the effect of these new compounds with previously investigated porphyrins, highlighting the structural and functional features in common and distinct among them. It allows to sketch a code for correlating their chemical structure to the interaction mechanism with 20S proteasome and, thus, to their modulatory properties.

## 2. Materials and Methods

### 2.1. Chemicals 

**Figure 1:**
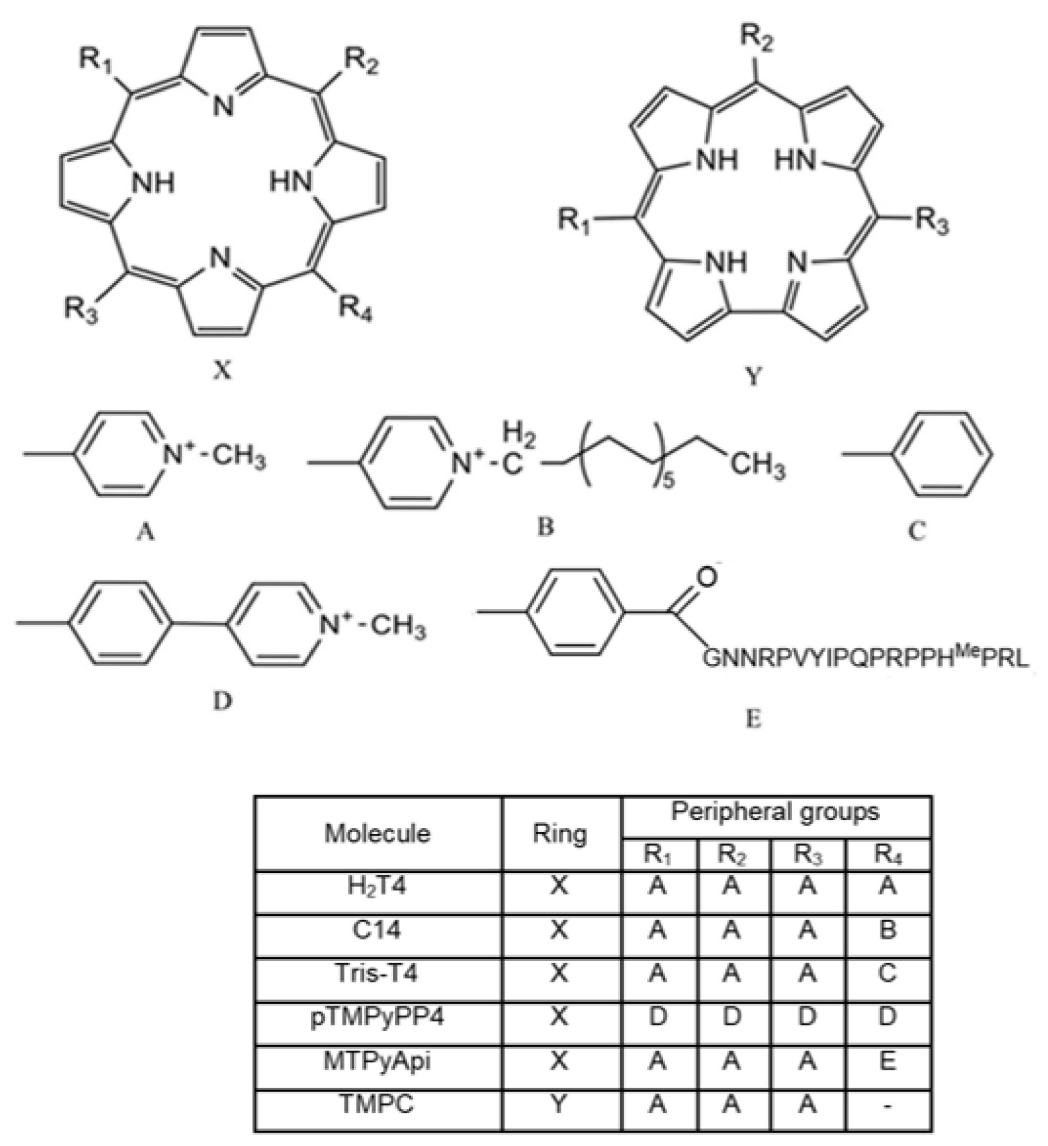
Porphyrin derivatives used in this work.

Purified human 20S proteasome (h20S) and the fluorogenic substrate (Suc-LLVY-AMC) were purchased from Boston Biochem (Cambridge, MA, USA). Meso-tetrakis(4-N-methylpyridyl)-porphine (H_2_T4) and its C_14_H_28_-alkyl derivative (C14) were obtained from Frontier Scientific Inc., Logan, UT, USA. Meso-tetrakis(4-N-methylphenyl pyridyl)-porphyrin (pTMPyPP4) and 5,10,15-Tri(N-methyl-4-piridyl) corrole (TMPC) were synthesized as previously reported [36]. 5-(phenyl)-10,15,20-tris(N-methyl-4-pyridyl)porphyrin (Tris-T4) was purchased from Midcentury (Posen, IL 60469, USA). The porphyrin derivative of Tris-T4 conjugated to the n-terminus of the antimicrobial peptide apidaecin (MTPyApi) was synthesized as reported elsewhere [34]. All porphyrin derivatives used in this work are reported in Figure 1.

### 2.2. Cell Culture

MCF-7 human breast cancer cells (ECACC, Sigma-Aldrich, St. Louis, MO 63118, USA) were maintained in Eagle’s minimum essential medium 1% non-essential amino acids (EMEM, Gibco, Thermofisher, Waltham, MA, USA) supplemented with 10% fetal calf serum (FCS, Gibco, Thermofisher, Waltham, MA, USA), 2mM glutamine (Sigma-Aldrich, St. Louis, MO 63118, USA), and 1% penicillin–streptomycin (Sigma-Aldrich, St. Louis, MO 63118, USA) at 37 °C in a humidified atmosphere with 5% CO_2_. Cultures were routinely split (1:6) at ~80% confluency by rinsing the cells with PBS without calcium or magnesium and released for sub-cultivation using 0.25% (*v*/*v*) trypsin-EDTA (Sigma-Aldrich, St. Louis, MO 63118, USA).

### 2.3. 20S Proteasomal Chymotryptic-like Activity Assays and Analysis of Kinetic Data

20S proteasome ChT-L activity assays were performed in the assay buffer (i.e., 50 mM TrisHCl, pH 8.0) by mixing 20S proteasome (2 nM) with increasing concentrations (0.1–10 μM) of MTPyApi and TMPC; mixtures were then incubated for 30 min at 37 °C. Afterwards, varying concentrations (between 5 μM and 100 μM) of fluorogenic peptide Suc-LLVY-AMC (specific for the ChT-L activity) were added in a 384 multi-well black plate. A minimum of three replicates were performed for each data point. The released AMC fluorescence was recorded at 440 nm (excitation at 360 nm) for 45 min, which is a time interval over which linearity was observed in a fluorescence plate reader (Varioskan, Thermo). In this investigation, we limited ourselves to the ChT-L activity for two main reasons, namely because (i) it is the most sensitive detectable activity in cell assays, and (ii) it is the most commonly employed enzymatical assay for proteasome and, thus, the most useful for comparison. Data relative to CP activities at different substrate concentrations have been analyzed by a double reciprocal Lineweaver–Burk plot, according to the following equation:

(1) $$\frac{\left[E_{0}\right]}{\nu }=\frac{K_{m}}{k_{cat}}\cdot \frac{1}{\left[S\right]}+\frac{1}{k_{cat}}$$

where (*E*_0_) is the enzyme concentration, *ν* is the observed velocity (expressed as moles of cleaved substrate per time interval unit), (*S*) is the substrate concentration, *K_m_* is the Michaelis–Menten equilibrium constant, referring to the affinity for substrate, and *k_cat_* is the velocity of the rate-limiting step. All curve fitting and statistical analyses were carried out using the non-linear fitting tool (NLFit) and MatLab (The Math works Inc., Natick, MA, USA). Parameters have been obtained by non-linear least-squares fitting of data; model discrimination and choice were based on the goodness of fit.

### 2.4. NMR Spectroscopy 

All NMR experiments were carried out at 298 K with an Inova 600 MHz spectrometer (Varian Inc., Palo Alto, CA, USA) equipped with a cryogenic probe that was optimized for ^1^H detection. As NMR binding assays are performed in aqueous buffer, the solubility of TMPC and MTPyApi in the solution of binding was tested, diluting the previously dissolved molecule in DMSO-d6 at the concentration of 175 μM in 50 mM HEPES (pH 7.6) and acquiring 1D ^1^H NMR spectra. At these concentrations, the MTPyApi spectra were poorly reproducible, impairing the analysis by NMR. On the other hand, TMPC solubility in aqueous solution was high enough to allow the NMR binding experiments to be carried out.

The NMR samples were prepared by dissolving unlabeled h20S protein at a concentration of 175 nM in aqueous buffer containing 50 mM HEPES, 100 mM NaCl, and 1mM DTT. STD spectra were acquired with 10,000 scans with on-resonance irradiation at 0.2 ppm for selective saturation of protein resonances and off-resonance irradiation at 30 ppm for reference spectra. A train of 40 Gaussian-shaped pulses of 50 ms with 1ms delay between pulses were used, for a total saturation time of 2 s. STD spectra were obtained by internal subtraction of the saturated spectrum from the reference spectrum by phase cycling with a spectral width of 7191.66 Hz, relaxation delay 1.0 s, 8 k data points for acquisition, and 16 k for transformation. The relative STD effect was calculated as the difference between the intensity (expressed as S/N ratio) of the highest peptide signal in the on-resonance STD spectrum and that in the off-resonance NMR spectrum, divided by the intensity of the same signal in the off-resonance spectrum: I_STD_ = I_0_ − I_sat_. Water LOGSY NMR experiments employed a 20 ms selective Gaussian 180° pulse at the water signal frequency and a NOE mixing time of 1 s. All NMR data were processed with the software VNMRJ 1.1.D (Varian Inc.). 1D spectra were analyzed by using the ChemAxon software (http://www.chemaxon.com, accessed on 8 June 2020).

### 2.5. Molecular Modeling 

Molecular modeling calculations were performed on SGI Origin 200 8XR12000 and E4 Server Twin 2 x Dual Xeon 5520, equipped with two nodes. Each node: 2 × Intel Xeon QuadCore E5520, 2.26 Ghz, 36 GB RAM. The molecular modeling graphics were carried out on a personal computer equipped with Intel(R) Core(TM) i7-4790 processor and SGI Octane 2 workstations.

#### 2.5.1. Calculation of the Chemical–Physical Properties of New Porphyrins

The apparent pK_a_ values were estimated by using the algorithm ACD/pK_a_ GALAS (ACD/Percepta software, version 2017.1.3, Advanced Chemistry Development, Inc., Toronto, ON, Canada, 2017, http://www.acdlabs.com, accessed on 19 July 2021). The new porphyrins TMPC and MTPyApi were considered in their prevalent cationic form in all calculations performed as a consequence of the estimation of percentage of neutral/ionized forms computed at pH 7.2 (cytoplasmic value) using the Handerson−Hasselbalch equation. The new porphyrins were built using the Insight 2005 Builder module (Accelrys Software Inc., San Diego, CA, USA). Atomic potentials were assigned using the CVFF force field, while the partial charges were assigned using the partial charges estimated by MNDO semi-empirical 1 SCF calculations [38]. The conformational space of porphyrins was sampled through 200 cycles of simulated annealing (SA; ε = 80 × r), followed by molecular mechanics (MM) energy minimization. During the SA procedure, the temperature is altered in time increments from an initial temperature to a final temperature by adjusting the kinetic energy of the structure (by rescaling the velocities of the atoms). The following protocol was applied: the system was heated to 1000 K over 2000 fs (time step of 1.0 fs); a temperature of 1000 K was applied to the system for 2000 fs (time step of 1.0 fs) to surmount torsional barriers; successively, temperature was linearly reduced to 300 K in 1000 fs with a decrement of 0.5 K/fs (time step of 1.0 fs). Resulting conformations were then subjected to MM energy minimization within Insight 2005 Discover 3 module (CVFF force field; ε = 80 × r) until the maximum rms was less than 0.001 kcal/Å using conjugate gradient [39] as the minimization algorithm. The resulting MM conformers were subsequently ranked by conformational energy (ΔE from the global energy minimum) and the interatomic distances between the charged nitrogen atoms.

A structural analysis on all the experimentally determined protein complexes containing apidaecin (PDB IDs: 5O2R, 4E81, and 4F00) was performed. The structures were downloaded from the Protein Data Bank (PDB; http://www.rcsb.org/pdb/, accessed on 16 March 2022) and analyzed by using the Macromolecules tool (Discovery Studio 2017 (Dassault Systemes BIOVIA, San Diego, CA, USA). In both structures, the apidaecin ligand assumed the same conformation. Starting from the global energy minimum of Tris-T4, a conformer of MTPyApi was built presenting the apidaecin moiety in the conformation found in the experimental structures. In particular, the first four amino acids were added in extended conformation by using the Build and Edit Protein tool (Discovery Studio 2017 (Dassault Systemes BIOVIA, San Diego). The resulting structure was merged with the proline-rich antimicrobial peptide Api137 bound to the ribosome (PDB ID: 5O2R) [40], and the residues Arg10 and His15 were replaced with Gln and 1-Methylhistidine, respectively (Macromolecules tool; Discovery Studio 2017). The global energy minimum of TMPC, the lowest energy conformer of MTPyApi presenting at least one free (solvent-accessible) face of the substituted porphyrin core, and the conformer of MTPyApi, presenting the apidaecin moiety in the extended conformation, were selected as input ligand structures for the subsequent docking studies.

#### 2.5.2. Docking Studies on Human 20S Proteasome

Docking calculations were performed by using our previously developed atomic molecular models of the closed and open conformations of full-length h20S [29]. According to our previous results [28,30], we selected for the docking studies the following starting points: (i) the substrate gate (closed state) for MTPyApi and (ii) the α1-α2, α4-α5, and α5-α6 grooves (closed and open states) for TMPC. In particular, in the case of MTPyApi, two different docking calculations were performed using the following starting complexes: (i) the lowest energy conformer of MTPyApi presenting at least one free (solvent-accessible) face of the substituted porphyrin core bound at the substrate gate (closed state) and (ii) the conformer of MTPyApi presenting the apidaecin moiety in the extended conformation bound at the substrate gate (closed state) through the porphyrin ring and at the α5-α6 groove through the apidaecin moiety as the analogue PR inhibitor co-crystallized with yeast 20S proteasome (y20S). On the other hand, in the case of TMPC, the docking calculations were performed using the following starting points: (i) one molecule at α5-α6 groove (closed state); (ii) one molecule at α4-α5 (open state); (iii) three molecules bound at α1-α2, α4-α5, and α5-α6 grooves (closed and open states). The putative starting complexes were subjected to dynamic docking studies (Affinity, SA_Docking; Insight2005, Accelrys, San Diego, CA, USA). In particular, a docking methodology, which considers all the systems flexible (i.e., ligand and protein), was used. Flexible docking was achieved using the Affinity module in the Insight 2005 suite, setting the SA Docking procedure [41] and using the cell multipole method for non-bonded interactions [42]. The docking protocol included a Monte-Carlo-based conformational search of the ligand within the obtained homology models of human 20S proteasome (i.e., closed and open conformation) for the random generation of a maximum of 20 acceptable complexes. During the first step, in the starting structures, the ligand was moved by a random combination of translation, rotation, and torsional changes to sample both the conformational space of the ligand and its orientation with respect to the binding domain area (MxRChange = 3 Å; MxAngChange = 180°). The binding domain area was defined as a subset including all residues of human 20S proteasome. Thus, all proteasome atoms were left free to move during the entire course of docking calculations, whereas, in order to avoid unrealistic results during the subsequent SA calculations, a tethering restraint was applied on the SCRs of the protein. The SCRs of the human 20S proteasome were identified using the Structure Prediction and Sequence Analysis server PredictProtein (http://www.predictprotein.org/, accessed on 21 July 2021). Within the identified SCRs, the following restraints were used: the distance between backbone hydrogen bond donors and acceptors in the alpha-helices was restrained within 2.5 Å. On the other hand, the φ and ψ torsional angles of the beta-sheets were restrained to −119° and +113°, or −139° and +135°, respectively, according to the presence of a parallel or anti-parallel structure. In particular, according to the reliability index values obtained from the secondary structure prediction, the following set of force constant values were applied (quadratic form): (i) 1 kcal/mol/Å^2^ (maximum force: 10 kcal/mol/Å^2^) for reliability index values from 0 to 3, (ii) 10 kcal/mol/Å^2^ (maximum force: 100 kcal/mol/Å^2^) for reliability index values from 4 to 6, and (iii) 100 kcal/mol/Å^2^ (maximum force: 1000 kcal/mol/Å^2^) for reliability index values from 7 to 9. During the Monte Carlo/Metropolis docking step, van der Waals (vdW) and Coulombic terms were scaled to a factor of 0.1 to avoid very severe divergences in the vdW and Coulombic energies. If the energy of a complex structure resulting from random moves of the ligand was higher by the energy tolerance parameter than the energy of the last accepted structure, it was not accepted for minimization. To ensure a wide variance of the input structures to be successively minimized, an energy tolerance value of 10^6^ kcal/mol from the previous structure was used. After the energy minimization step (conjugate gradient; 2500 iterations; ε = 1), the energy test, with an energy range of 50 kcal/mol, and a structure similarity check (rms tolerance = 0.3 kcal/Å) was applied to select the 20 acceptable structures. Each subsequent structure was generated from the last accepted structure. Following this procedure, the resulting docked structures were ranked and analyzed considering the nonbonded interaction energies between the ligand and the enzyme (vdW and electrostatic energy contribution; group-based method [43]; CUT_OFF = 100; ε = 2 × r; Discover_3 Module of Insight2005). The Monte Carlo docked complexes were then subjected to molecular dynamics simulations at flexible temperatures (Simulated Annealing, SA) to enhance the fixing of the ligand into the binding site and to explore possible ligand-induced large-scale conformational changes of the protein. In particular, the resulting docked complexes were subjected also to a molecular dynamics SA protocol using the Cell_Multipole method for non-bonded interactions and the dielectric constant of the water (ε = 80 × r). A tethering restraint was applied on the SCRs of the complex. The set of structural restraints applied was the same as for previous docking calculations. The protocol included 5 ps of a dynamic run divided in 50 stages (100 fs each) during which the temperature of the system was linearly decreased from 500 to 300 K (Verlet velocity integrator; time step = 1.0 fs). In simulated annealing, the temperature is altered in time increments from an initial temperature to a final temperature. The temperature is changed by adjusting the kinetic energy of the structure (by rescaling the velocities of the atoms). Molecular dynamics calculations were performed using a constant temperature and constant volume (NVT) statistical ensemble and the direct velocity scaling as temperature control method (temp window = 10 K). In the first stage, initial velocities were randomly generated from the Boltzmann distribution, according to the desired temperature, while, during the subsequent stages, initial velocities were generated from dynamics restart data. The temperature of 500 K was applied with the aim of surmounting torsional barriers, thus allowing an unconstrained rearrangement of the “ligand” and the “protein” binding site (initial vdW and Coulombic scale factors = 0.1). Successively temperature was linearly reduced to 300 K in 5 ps, and, concurrently, the vdW and Coulombic scale factors have been similarly increased from their initial values (0.1) to their final values (1.0). A final round of 10^5^ minimization steps (ε = 80 × r) followed the last dynamics steps, and the minimized structures were saved in a trajectory file. The ligand/enzyme complexes thus obtained were ranked by their conformational energy and analyzed considering the non-bonded interaction energies between the ligand and the enzyme (vdW and electrostatic energy contribution; group-based method; CUT_OFF = 100; ε = 2 × r; Discover_3 Module of Insight 2005). The resulting complexes, showing the best non-bonded interaction energy either after the Monte Carlo/Metropolis procedure or after the dynamic simulation (SA) were selected as representatives of the most probable porphyrin binding modes. The quality of the selected complexes was then checked using MolProbity structure evaluator software [44] and compared to that of the reference PDB structure.

Solvent accessible surface (SAS; H_2_O probe) calculations were performed using Discovery Studio 2017 (Dassault Systèmes BIOVIA, San Diego, CA, USA, 2017). The overall SAS and that of each hydrogen atom of the TMPC molecules bound to h20S in the selected docked complexes (starting either from the closed or the open protein conformation) were calculated. Then, for the hydrogen atoms equivalent in NMR experiments, a SAS average value was calculated. The resulting values were compared to the SAS values of the unbound molecule. The rate of SAS decrease was calculated by using Microsoft Excel.

### 2.6. Cell Viability Assay and Proteasome-GloTM Cell-Based Assay 

Cell viability was determined by the colorimetric MTT (3-(4,5-Dimethylthiazol-2-yl)-2,5-Diphenyltetrazolium Bromide, Invitrogen) metabolic activity assay. The cells were seeded in a 96-well plate at a density of 5 × 10^3^ cells/well and exposed at 10 μM of porphyrins in the dark for 48 h. Then, cells were incubated in a 0.5 mg/mL MTT solution (in EMEM) for 4 h at 37 °C; the intensity of the formazan produced from enzyme cleavage of the tetrazolium salt by metabolically active cells is proportional to the number of viable cells. The medium was removed and DMSO was added to solubilize the MTT formazan crystals. The plate was mixed for 30 min and the absorbance (570 nm) was measured using a plate-reader (Varioskan Thermo). The data are three replicates expressed as the percentage of MTT reduction regarding the untreated cells (positive control). The chymotrypsin-like proteolytic activity of the proteasome in intact MCF7 cells was determined using the bioluminescent Proteasome-Glo^™^ cell-based assay system (G8660; Promega, Mannheim, Germany) according to manufacturer’s instructions. Briefly, 800 cells/well were cultivated in 384 sterile multi-wells (optical plate and white walls) in 50 µL of EMEM + FCS 10% + penStrep. After cell seeding (6 h), porphyrins were added in the cell medium at 10 µM concentration for 24 h. After treatment, attached cells were washed twice with PBS and relative ChT-L activity was determined. The substrate for the ChT-L activity (Suc-LLVY-aminoluciferin) was dissolved in Proteasome-Glo™ cell-based reagent and added to intact cells (1/1 vol). After 2 min of shaking for permeabilization and further 10 min of incubation, luminescence was measured with a luminometer (Varioskan Flash, Thermo). Each sample was analyzed in quadruplicate. Data are reported as normalized means, setting the untreated cells to 100%. ± SD. Analysis of variance has been performed by one-way ANOVA followed by statistical Sidak’s test.

## 3. Results and Discussion

### 3.1. Structural Features of the New Ligands 

In order to expand the structure–activity relationships (SARs) obtained by our previous studies on porphyrin-based proteasome modulators [28,30], we investigated the behavior of the porphyrin–apidaecin hybrid MTPyApi and the corrole derivative TMPC. Indeed, MTPyApi will provide useful information on the role played by the peripheral substituents in regulating ligand binding modes and, in particular, the role of charge distribution in modulating the h20S interaction with substrates. On the other hand, to unveil the role played by the porphyrin core, this was replaced with a corrole scaffold, a “contracted” version of the tetrapyrrolic ring due to the lack of a methine bridge. Accordingly, TMPC lacks one substituent compared to the tetra-substituted porphyrin derivatives (Figure 1).

From a structural point of view, MTPyApi might be considered as a Tris-T4 molecule, where the phenyl group has been covalently linked to the N-terminus of apidaecin. However, the presence of the long and amphipathic apidaecin tail renders MTPyApi quite a complex molecule in terms of its conformational properties and charge distribution [45]. This aspect emerges also in the molecular modeling of these molecules. The calculated pK_a_ values and prevalent ionic forms at the physiological pH (7.2) of MTPyApi and TMPC are reported in Table S1. We obtained, as prevalent, the tri-cationic form for TMPC (92%; comparable to Tris-T4 [30]) and the hexa-cationic form for MTPyApi (80%), respectively. In fact, the apidaecin tail presents an uneven distribution of three additional positive charges: one in the central portion, a second charge toward the end of the sequence, and another one located in the proximity of the N-terminal end attached to the porphyrin core (Figure 1). Their 3D spatial distribution strongly depends on the conformation of the peptide conjugated to the porphyrin core.

**Figure 2:**
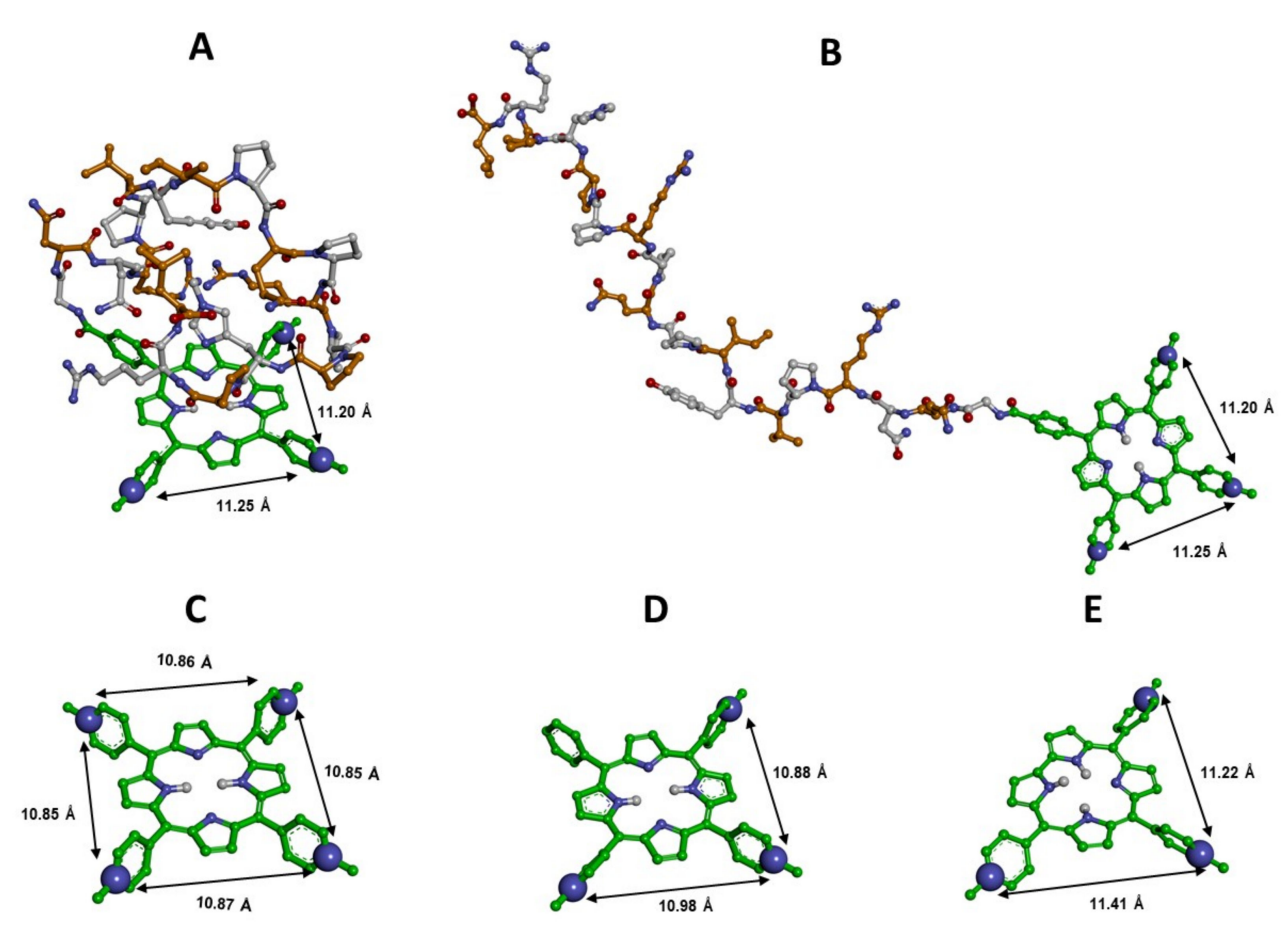
Comparison of the calculated structures of MTPyApi (**A**,**B**) and TMPC (**E**) with those of the reference compounds H_2_T4 (**C**) and Tris-T4 (**D**). The inter-atomic distances between adjacent protonated nitrogen atoms are reported. Molecules are displayed in ball-and-stick with the pyridine nitrogen atoms evidenced in CPK (scaled by 50%). The porphyrin ring carbon atoms are colored in green. Carbon atoms of the apidaecin residues are colored in white and orange alternately. For sake of clarity, only the hydrogen atoms of the pyrrole nitrogen are displayed.

Then, the molecular models of the two ligands in their prevalent ionic form were built and subjected to conformational analysis (see the Experimental Section in Supplementary Materials for details). All the resulting conformers showed a distance of ~11 Å between the charged nitrogen atoms of adjacent N-methyl pyridine rings, as also found for H_2_T4 [28] and Tris-T4 [30]. However, some structural differences are present in the new ligands as compared to the reference compounds (Figure 2).

In particular, MTPyApi is characterized by the presence of the antimicrobial peptide apidaecin attached via an amide linker at the para position of the phenyl substituent of the porphyrin ring. Our conformational analysis, performed implicitly mimicking the polarity of a water medium (ε = 80 × r), indicated that, in the lowest energy conformers of MTPyApi, the peptide apidaecin is folded on the porphyrin core. However, the structural analysis, performed on all the experimentally determined protein complexes containing apidaecin peptides as ligands (i.e., with ribosome (PDB ID: 5O2R) [40] and with the chaperone DnaK (PDB IDs: 4E81 and 4F00) [46,47]), show that the apidaecin tail always assumes the same extended conformation. Therefore, we also modeled the conformer of MTPyApi, presenting the apidaecin moiety in the same extended conformation (see Section 2 for details). Both the lowest energy conformer of MTPyApi, presenting at least one free (solvent-accessible) face of the substituted porphyrin core, and the conformer of MTPyApi, presenting the apidaecin moiety in the extended conformation (all reported in Figure 2A,B), were selected as input ligand structures for the subsequent docking studies (see below).

On the other hand, in TMPC, there is only one global energy minimum (Figure 2E), where three aromatic substituents are present on the pyrrole ring system and the more constrained corrole ring displays an additional hydrogen atom on the pyrrole core, thus deviating from planarity with respect to the porphyrin ring (Figure S1).

### 3.2. Proteasome Assays on the Isolated 20S CP

Starting from this structural analysis, the observed differences turn out to be crucial when the functional effect of these derivatives on the h20S ChT-L activity is evaluated. As a matter of fact, the presence of the peptidic chain appears to heavily reverse/disrupt the interaction mechanism, induced by Tris-T4 [30], transforming MTPyApi into a simple competitive inhibitor, such as the tetracationic H_2_T4, which has already been investigated [28]; therefore, we found it more useful to compare the MTPyApi behavior with that of H_2_T4. On the other hand, the corrole ring of TMPC displays a tri-cationic decoration, as with Tris-T4 (see Figure 1 and Figure 2), and this might account for the qualitatively similar behavior (see below), which justifies their functional comparison.

#### 3.2.1. Comparison of H_2_T4 and MTPyApi

**Figure 3:**
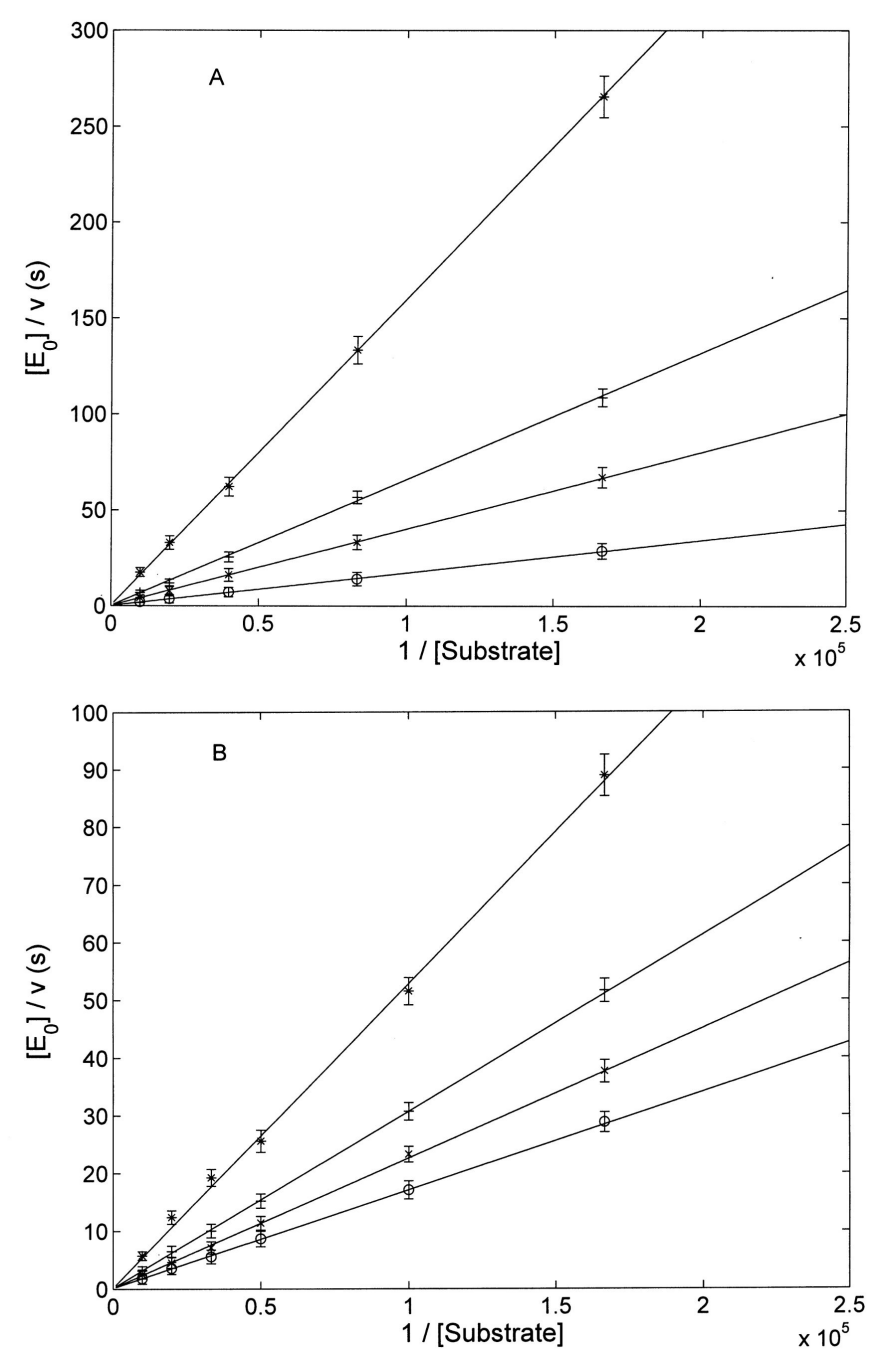

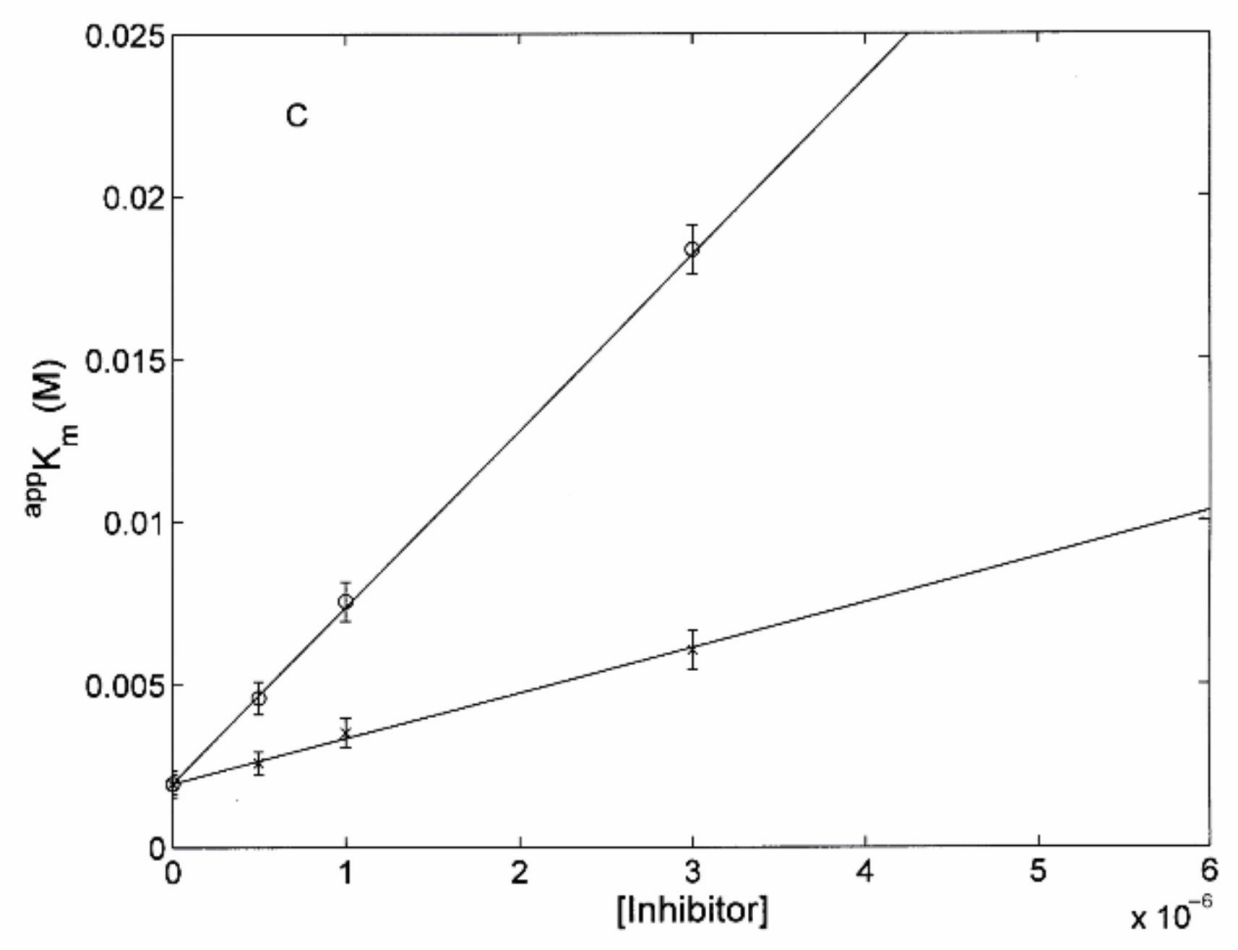
(**A**) Lineweaver–Burk double-reciprocal plot as a function of substrate concentration of h20S ChT-L activity in the absence (o) and in the presence of 0.5 µ (×), 1 µM (+), and 3 µM (*) H_2_T_4_. Continuous lines are the non-linear least-squares fitting of data according to Equation (1). (**B**) Lineweaver–Burk double-reciprocal plot as a function of substrate concentration of h20S ChT-L activity in the absence (o) and in the presence of 0.5 µM (×), 1 µM (+), and 3 µM (*) MTPyApi. Continuous lines are the non-linear least-squares fitting of data according to Equation (1). (**C**) Dependence of *K_m_* on the concentration of H_2_T4 (o) and MTPyApi (×). Continuous lines are the non-linear least-squares fitting of data according to Equation (2).

Both H_2_T4 and MTPyApi show a competitive inhibition behavior with respect to the ChT-L activity of h20S (Figure 3). However, the different concentration dependence of their K_m_ (Figure 3C) might be related to their different structure.

From the slope of the dependence of K_m_ on porphyrin concentration, we can calculate the value of the competitive inhibitor affinity constant K_I_ according to the following Equation (2)

(2) Kmapp=Km0⋅(1+IKI)

where *^app^K_m_* is the value of *K_m_* at a given concentration I of porphyrin, ^0^*K_m_* is the value of *K_m_* in the absence of porphyrins, [*I*] is the porphyrin concentration, and *K_I_* is the value of the inhibitory constant. In particular, H_2_T4 shows a value of *K_I_* = 3.6 (±0.5) × 10^−^^7^ M, whereas MTPyApi shows a *K_I_* = 1.4 (±04) × 10^−^^6^ M (Figure 3C). Recalling that *K_I_* refers to the dissociation process, it is immediately evident that the affinity of H_2_T4 versus h20S is fourfold higher than that measured for MTPyApi. Most likely, this difference can be explained by the lack in MTPyApi of a positively charged N-methyl-pyridine ring and the concomitant presence of the long peptide chain, weakening the electrostatic interaction with the gate, as compared to H_2_T4 (see Section 2.5).

#### 3.2.2. Modulatory Properties of TMPC and Its Comparison with Tris-T4

These two porphyrins share the peculiarity of modulating the h20S inhibition/activation depending on their concentration.

**Figure 4:**
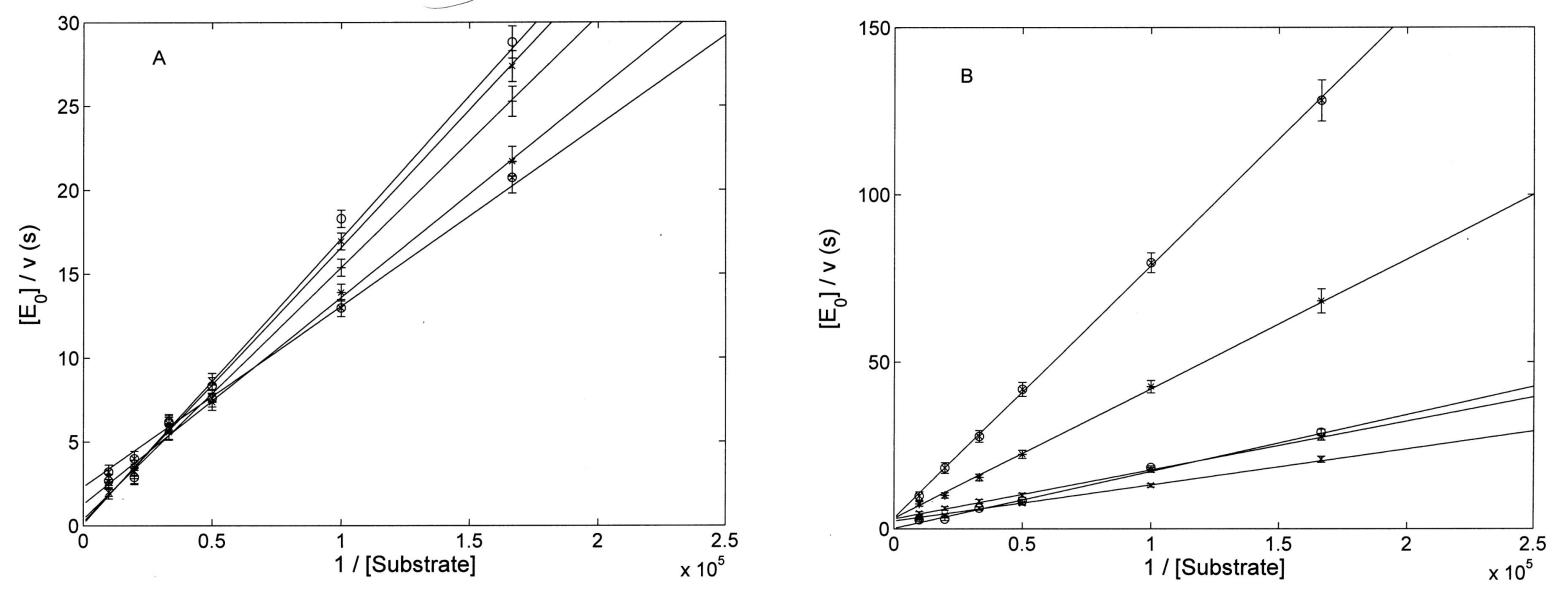
(**A**) Lineweaver–Burk double reciprocal plot as a function of substrate concentration of h20S ChT-L activity at different concentrations of TMPC, namely 0 (o), 0.1 μM (×), 0.15 μM (+), 0.3 μM (*), and 0.5 μM (⊗). Continuous lines are the non-linear least-squares fitting of data according to Equation (1). (**B**) Lineweaver–Burk double reciprocal plot as a function of substrate concentration of h20S ChT-L activity at different concentrations of TMPC, namely 0 (o), 0.5 µM (×), 1.0 μM (+), 3.0 μM (*), and 6.0 μM (⊗). Continuous lines are the non-linear least-squares fitting of data according to Equation (1).

Figure 4 shows the Lineweaver–Burk plot of the effect on the ChT-L activity of h20S by TMPC. The activity of TMPC as an activator of the ChT-L activity of the h20S for [TMPC] ≤ 1 µM is evident from the data reported in Figure 4A. As anticipated, at higher porphyrin concentrations, TMPC shows a completely different effect, behaving as a competitive inhibitor (Figure 4B).

**Figure 5:**
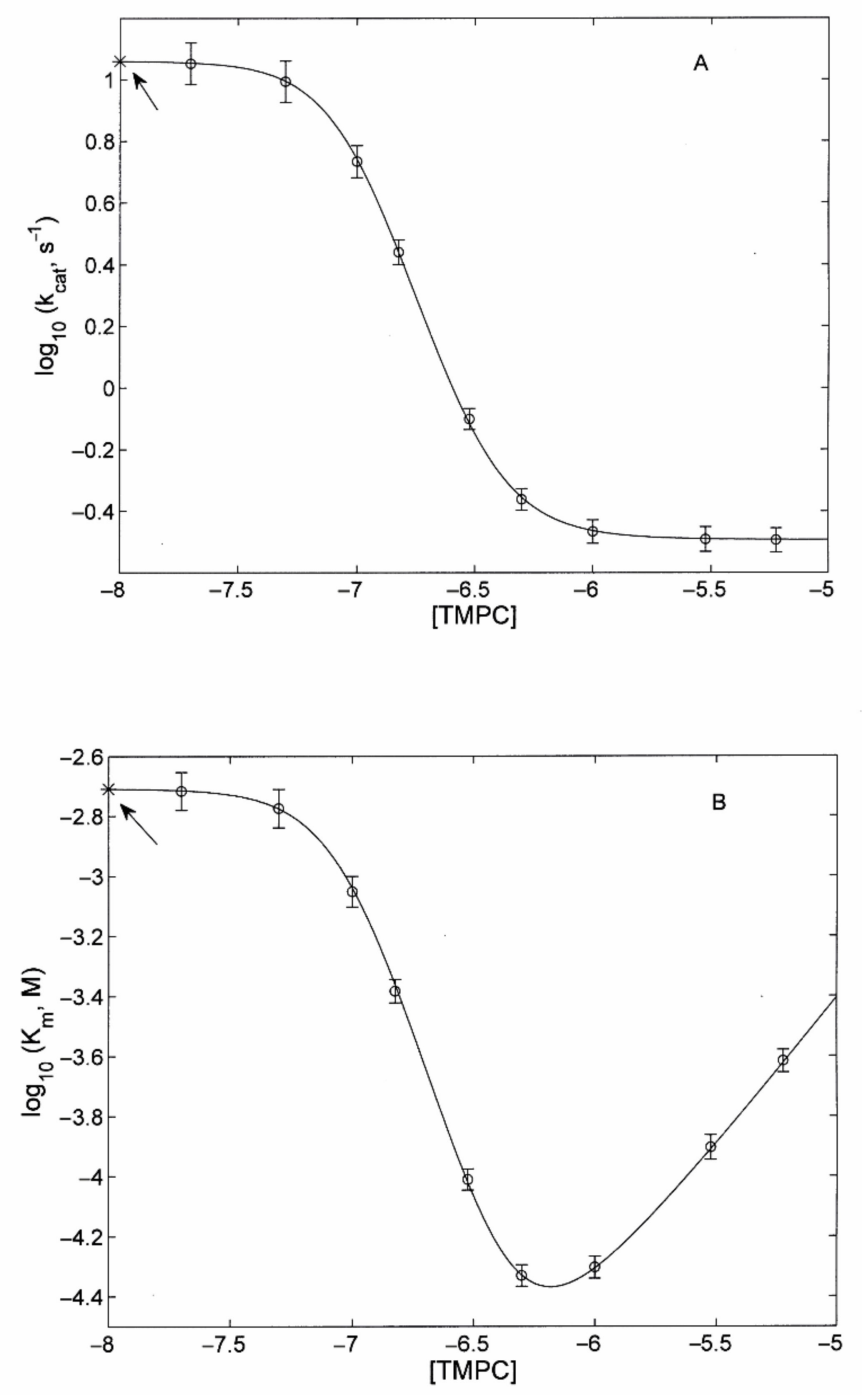

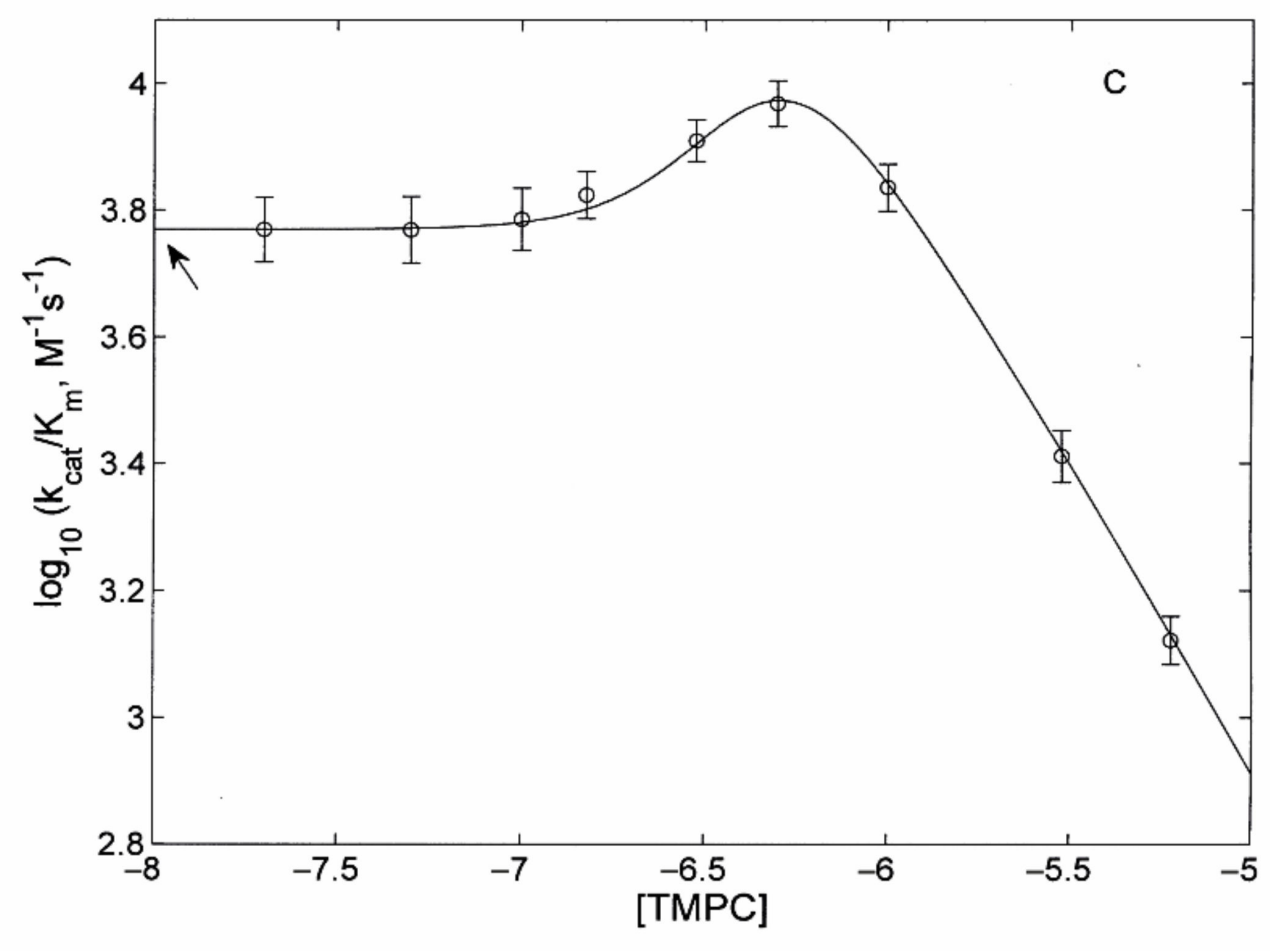
Dependence of *k_cat_* (**A**), *K_m_* (**B**), and *k_cat_*/*K_m_* (**C**) for h20S proteasome ChT-L activity on TMPC. Continuous lines are obtained by non-linear least-squares fitting of data according to Equation (3a–c), respectively, employing parameters reported in Table 1. Arrows indicate the values of catalytic parameters in the absence of TMPC and asterisks correspond to the measured values of *k_cat_* and *K_m_*.

Altogether, these pieces of evidence suggest that, unlike H_2_T4 and MTPyApi, TMPC induces (similarly to Tris-T4 [30]) a structural change in the h20S toward a conformational state that is characterized by a higher enzymatic activity and a slower rate-limiting step (i.e., *k_cat_*) (as suggested by the behavior observed in Figure 4A). However, for [TMPC] ≥ 0.5 µM, it interacts (most likely) with an additional site, whose occupancy affects the substrate binding to the catalytic site (see Figure 4B). This complex behavior results in a peculiar dependence of catalytic parameters on [TMPC] (see Figure 5).

**Scheme 1:**
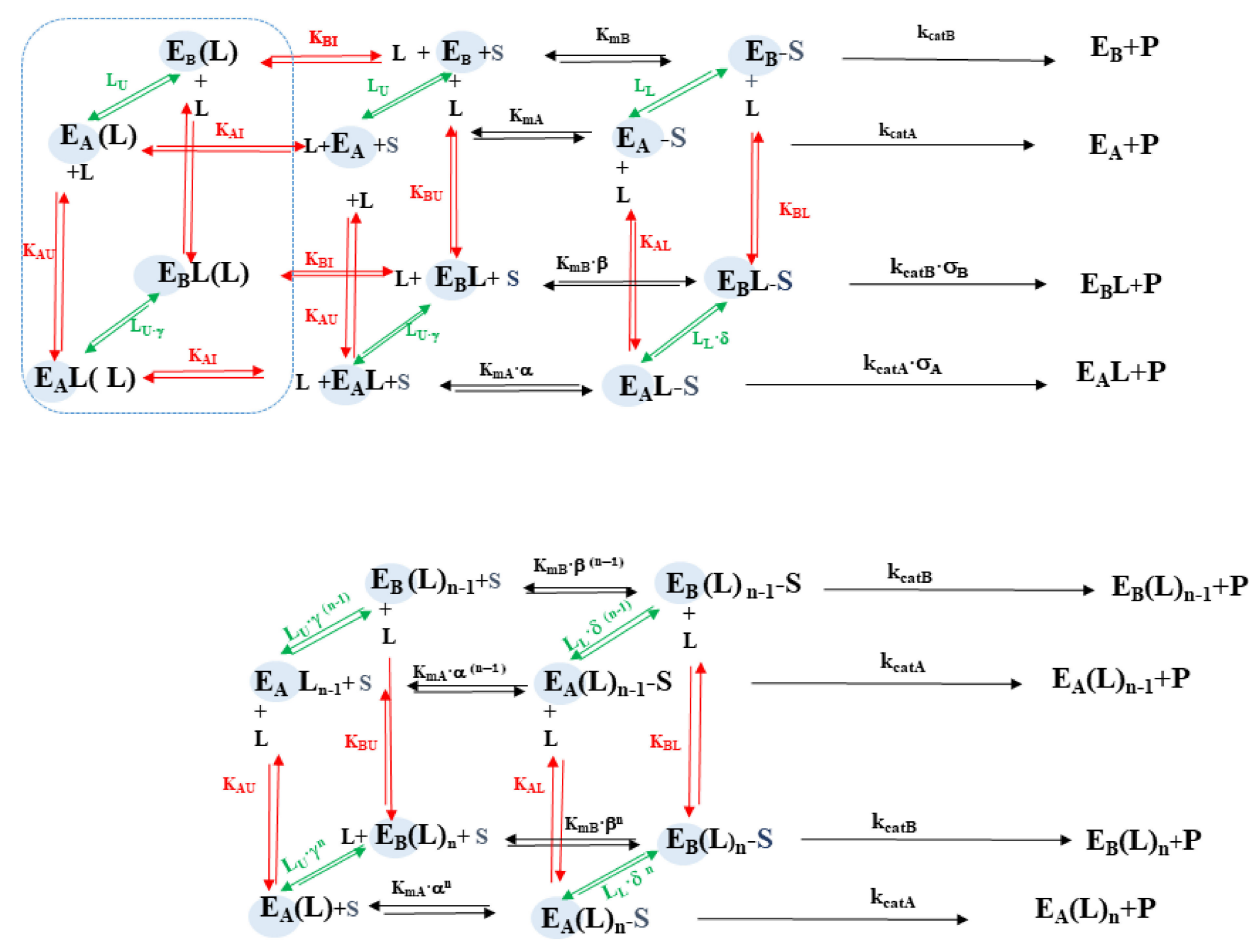
Thermodynamic description of equilibria involved in the modulation of enzymatic activity of h20S (E) on the substrate (S) by the porphyrin TMPC. In the upper part is the scheme for the first TMPC binding step, while in the lower part is the scheme for the last n-th TMPC binding step. The left cube (enclosed in the blue rectangle) concerns the binding of TMPC to the competitive inhibitory site, which, for the sake of clarity, is depicted only for the first binding step, but it may occur also at all intermediate bound degrees, concerning only the substrate-free enzyme. Parameters concerning the enzymatic activity are depicted in black, those regarding TMPC binding are depicted in red, and conformational equilibria are depicted in green. The meaning of various parameters is: *k_catA_* and *k_catB_* are the rates of the rate-limiting step in the two states A and B, *K_mA_* and *K_mB_* are the Michaelis–Menten constants in A and B, *K_AU_* and *K_BU_* are the affinity constant for TMPC of the 20S proteasome in the absence of substrate in the two states A and B, *K_AL_* and *K_BL_* are the affinity constant for TMPC of the 20S proteasome in the substrate-bound ES complex, while *K_AI_* and *K_BI_* are the affinity constant for TMPC to the inhibitory competitive site of the 20S proteasome. *L_U_* is the equilibrium constant between A and B in the absence of the substrate, *L_L_* is the equilibrium constant between A and B in the substrate-bound ES complex, and “n” is the interaction parameter, which reflects the minimum number of porphyrin units binding cooperatively to their site on h20S.

Such a complex behavior requires a multidimensional approach, as reported in Scheme 1. The analysis of data reported in Figure 5A–C is based, indeed, on the thermodynamic framework reported in Scheme 1.

In addition, for the sake of clarity, in Scheme 1, some of these parameters are reported as interaction ratios, namely α = *K_AU_*/*K_AL_*, β = *K_BU_*/*K_BL_*, γ = *K_BU_*/*K_AU_*, δ = *K_BL_*/*K_AL_*. Therefore, the dependence of catalytic parameters, reported in Scheme 1 and described by continuous lines in Figure 5A–C, has been carried out employing Equation (3a–c)

(3a) Kcatobs=kcatA⋅(1+KAL⋅TMPC)n+LL⋅kcatB⋅(1+KBL⋅TMPC)n(1+KAL⋅TMPC)n+LL⋅(1+KBL⋅TMPC)n

(3b) Kmobs=Km0⋅(1+KAI⋅TMPC)m⋅(1+KAU⋅TMPC)n+LU⋅(1+KBI⋅TMPC)m⋅(1+KBU⋅TMPC)n(1+KAL⋅TMPC)n+LL⋅(1+KBL⋅TMPC)n

(3c) kcatobsKmobs=kcatAKmA⋅(1+KAI⋅TMPC)m⋅(1+KAU⋅TMPC)n+LU⋅kcatBKmB⋅(1+KBI⋅TMPC)m⋅(1+KBU⋅TMPC)n(1+KAU⋅TMPC)n+LU⋅(1+KBU⋅TMPC)n

where ^o*bs*^*k_cat_*, *^obs^K_m_*, *^obs^k_cat_*/*^obs^K_m_* are the observed catalytic parameters at a given concentration of TMPC, ^0^*K_m_* corresponds to *K_mA_*, “n” is the number of concerted allosteric binding sites for TMPC, and “m” is the number of competitive inhibitory sites. In the case of h20S, the minimum value to account for the quite steep dependence of catalytic parameters turns out to be n = 3 and m = 1 for TMPC, as reported in Figure 5A–C.

The occurrence of a concerted activation mechanism, envisaging the interaction of three porphyrin molecules (supported also by computer simulations), is accompanied at higher TMPC concentrations by a competitive inhibitory mechanism. It represents, so far, a unique property of this porphyrin, underlying the potentiality for TMPC of playing a double role as a proteasome activator (in the nanomolar range, up to 0.5 µM) and inhibitor (in the micromolar range, above 1 µM) according to which concentration range is employed. This behavior shows similarities and, at the same time, differences from that of Tris-T4 [30]. To obtain an immediate visual comparison, we also report in Table 1 the data for Tris-T4 (please note that they refer to the same mechanism described in Scheme 1 except for competitive inhibitory activity). Considering that, in the analysis of the behavior reported in Figure 5A–C, some parameters (i.e., *k_catA_*, *K_mA_*, *L_U_*, and *L_L_*) have been imposed equally for both porphyrins (and, consequently, also *k_catB_* and *K_mB_*) since they are substrate-linked but porphyrin-ligand-independent, a comparison between the two porphyrins allows several considerations to be made:

(a) both porphyrins appear to activate the h20S by inducing a conformational transition from the A to the B functional state through a cooperative action, involving, in both cases, a clustering of (at least) three porphyrin molecules;
(b) the main determinant of the higher affinity of TMPC as a proteasome activator is the binding of the porphyrin to the substrate-bound A state (with an almost thirty-fold larger binding constant *K_AL_* with respect to Tris-T4; see Table 1);
(c) in the B state, binding constants for TMPC are only moderately (two- to three-fold) higher than for Tris-T4 (see Table 1);
(d) in the B conformation of h20S TMPC shows an additional binding site (not detected for [Tris-T4] ≤ 10 µM; see Ref. [30]), characterized by *K_BI_* (see Table 1), where it exerts a competitive inhibitory activity, which, apparently, reverts the activation effect;
(e) although an inhibitory site for Tris-T4 had been detected only in y20S, its affinity was much lower than that observed here for TMPC in h20S (see Table 1 and Ref. [30]); in y20S the value of *K_AI_* for Tris-T4 was very low (i.e., *K_AI_* ≤ 10^4^ M^−^^1^), so it could be ignored. In the case of TMPC, *K_AI_* (= 10^6^ M^−^^1^; see Table 1) is instead large enough to play a significant role, and we had to take it into account.

### 3.3. Interaction of TMPC by NMR Spectroscopy

**Figure 6:**
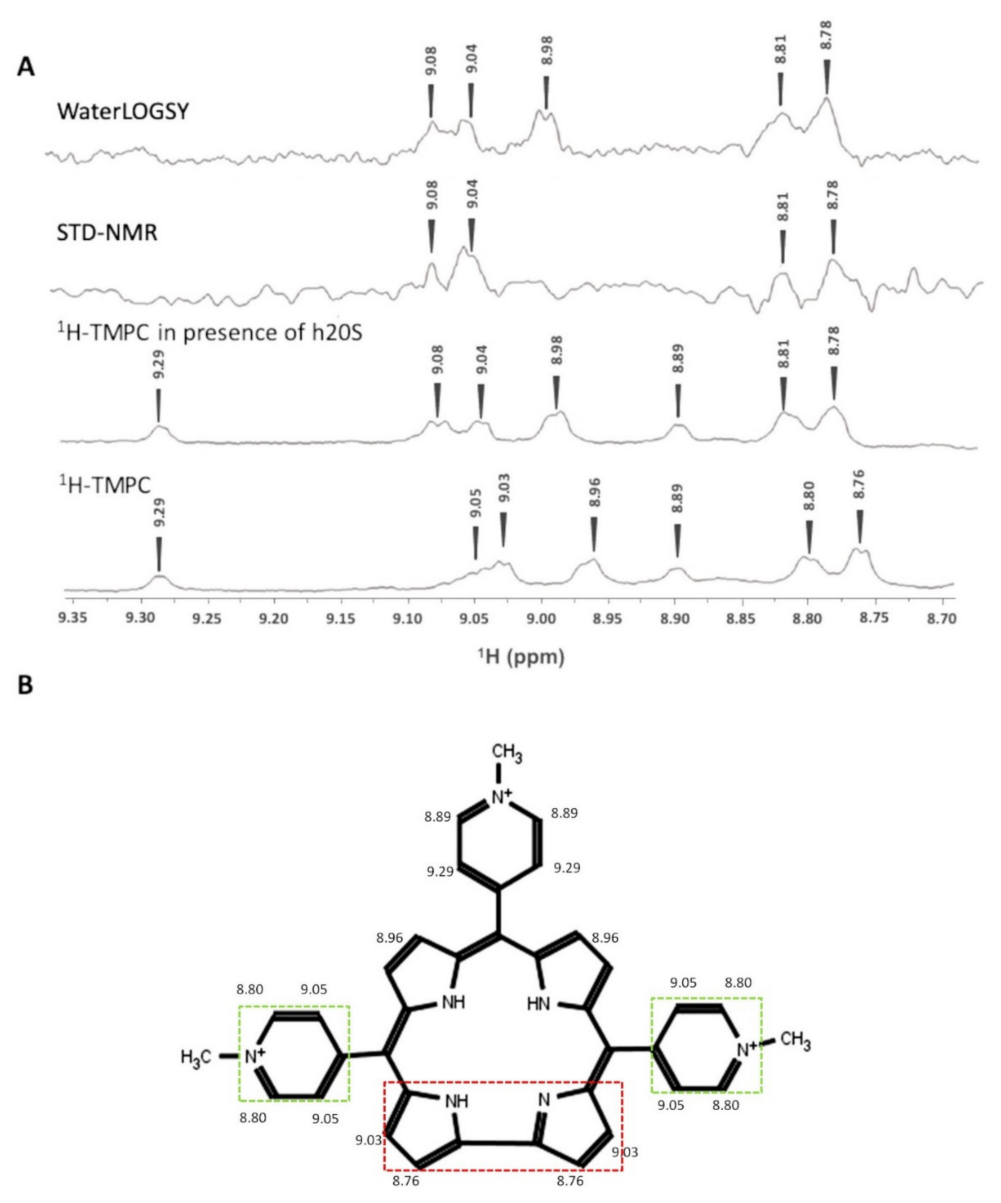
(**A**) Low-field of ^1^H NMR spectrum of TMPC (175 μM) in the buffer (50 mM Hepes, 100 mM NaCl, and 1mM DTT) and in the presence of h20S proteasome (860 nM); STD-NMR and WaterLOGSY spectra (from the bottom to the top). (**B**) Structure of the TMPC reporting ^1^H chemical shift assignments and highlighting the ^1^H TMPC resonances visible in STD (broken line in red and in green) and in WaterLOGSY (broken line in red) spectra.

In order to provide structural information on the molecular recognition mechanism of the binding between TMCP and h20S, NMR-ligand-based observations, such as STD-NMR and WaterLOGSY experiments, have been performed. 1D ^1^H NMR spectra have been used for the assignment of the ^1^H chemical shifts of the molecule. As shown in Figure 6, STD-NMR and WaterLOGSY spectra of TMPC, in the presence of a sub-stoichiometric amount of h20S, clearly indicate that the ligand interacts with the h20S and allows to identify the TMPC moiety more affected by the protein interaction [48,49]. In particular, aromatic signals of the directly bound pyrroles, together with adjacent methyl-pyridine rings, are clearly visible in the STD-NMR spectrum and, therefore, more closely involved in the h20S binding interaction. The same result was obtained by means of WaterLOGSY spectra, except for the positive signal of the para-methyl pyridine rings, which are not visible due to a low signal-to-noise ratio of the spectra.

The outcomes of the NMR spectroscopic investigation clearly reveal the TMPC region in close contact with the h20S surface, which are those surrounded by broken lines (see Figure 6B). However, the low signal-to-noise ratio of the STD and WaterLOGSY spectra led us to not exclude that the remaining part of the molecule also interacts with the protein, although to a lesser extent.

### 3.4. Docking Studies

Since the X-ray structure of the complexes between h20S and porphyrin-based ligands cannot be experimentally determined due to the formation of ligand aggregates, the SARs, sketched by this study, have been investigated in silico. Dynamic docking studies were performed using as protein structures the previously developed atomic molecular models of full-length h20S (closed and open conformations) [29]. Although, during the docking procedure, the entire h20S structure was dynamically explored as a possible ligand binding site, nevertheless, the calculation requires a reasonable starting complex. We previously showed how cationic porphyrins can bind to the surface of h20S proteasome, exploiting the interaction with the negatively charged residues involved in proteasome activation by RPs, which form a sort of electrostatic key code for the allosteric modulation of the 20S catalytic activities [28,30]. In particular, while the competitive antagonist H_2_T4 was calculated to bind at the substrate gate, the allosteric modulator Tris-T4 was predicted to bind at the grooves between the α-subunits. Accordingly, based on the observed inhibition mechanism of the newly discovered h20S ligands, we applied to MTPyApi and TMPC the same docking procedure and used the same starting complexes previously reported for H_2_T4 [28] and Tris-T4 [30], respectively. Therefore, we placed the competitive porphyrin MTPyApi at the substrate gate (closed state) and the allosteric modulator TMPC at the α1-α2, α4-α5, and α5-α6 grooves (closed and open states). In particular, in the case of TMPC, different docking calculations were performed using the following starting complexes: (i) one molecule bound at the α5-α6 groove (closed state); (ii) one molecule bound at α4-α5 (open state); (iii) three molecules bound at the α1-α2, α4-α5, and α5-α6 grooves (closed and open states).

In order to simulate the dynamics of the ligand–protein complex formation, we applied an original docking protocol based on a Monte Carlo/Metropolis search (first molecular recognition event) followed by molecular dynamics simulations at flexible temperatures (SA) (fixing of the ligand into the binding site and exploration of possible ligand-induced protein large-scale conformational changes)**.** During the Monte-Carlo-based simulation, the ligand was randomly moved, including either conformational changes and translation and rotation around the protein, such that a different region of the search space (the whole h20S structure) was sampled at each step. The generated structures were energy-minimized and then selected following energetic and probabilistic acceptance criteria. The resulting complexes were subjected to the SA procedure, and the entire complex structure was considered flexible during all calculations. Hence, in order to avoid unrealistic results, conformational constraints were applied to the backbone atoms of the conserved secondary structures (Table S2) and the structural quality of the calculated complexes was assessed by using the MolProbity structure evaluator software [44] (Table S3; see Section 2 for details). Among the resulting complexes (Figures S2–S7 and Tables S4–S9), those showing the best non-bonded interaction energies were selected as representatives of the most probable ligand binding modes (best-docked solutions).

#### 3.4.1. Docking of MTPyApi

Due to the high conformational variability of apidaecin peptides and their reported conformational changes according to different chemical–physical environments [45], we performed two different docking simulations using two different starting conformations of MTPyApi. In one, the peptide tail establishes interactions with the porphyrin ring, as resulted by our conformational analysis, while, in the other one, the extended conformation was adopted, as observed for apidaecin in complex with different protein partners (Figure 2A,B, respectively). It has to be underlined that the ligand conformation is, notwithstanding, fully explored during the docking simulation; however, we took into account that, in the case of such a complex ligand and large interacting protein, the starting conformation could affect the docking results.

**Figure 7:**
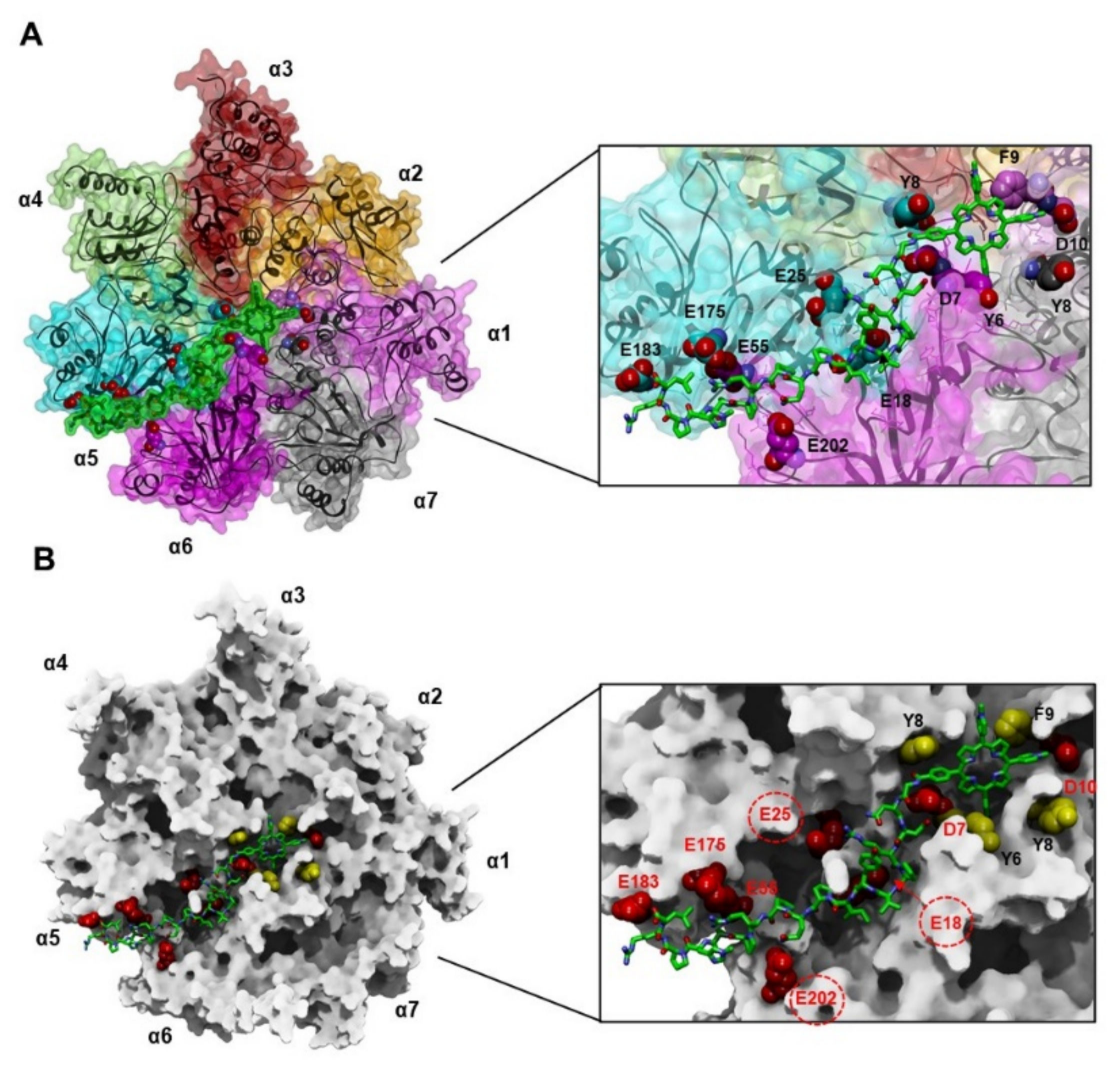
Selected docked complex of MTPyApi bound to h20S (closed conformation). (**A**,**B**): top and close-up view of the ligand binding site. The negative residues interacting with MTPyApi and those in common with H_2_T4 (porphyrin moiety) are displayed in CPK. (**A**) h20S backbone is displayed as black solid ribbons, while the transparent solvent accessible surface (SAS) and the displayed residues are colored in pink (α1), orange (α2), brown (α3), light green (α4), cyan (α5), magenta (α6), and gray (α7). MTPyApi is colored by atom type (C: black; N: blue; O: red), and its SAS is displayed (green). (**B**) h20S SAS is displayed as solid and colored in white. MTPyApi is colored by atom type (C: green; N: blue; O: red) and displayed in stick. The h20S interacting residues are colored: negatively charged = red, aromatic = yellow. The glutamate residues involved in ionic interactions with RPs are evidenced with a red dashed circle.

Starting from both MTPyApi conformations, the best-docked structure shows the porphyrin moiety bound to the substrate gate, as found for H_2_T4, the two compounds sharing the interactions with negatively charged and aromatic residues, some of them also involved in key ionic interactions with RPs (see Figure 7and Figure S8). However, according to the lack of a positively charged N-methyl-pyridine substituent, the porphyrin moiety of MTPyApi is not able to reproduce the planar tetra-cationic pharmacophore of H_2_T4 (Figure 2A–C) and, by consequence, to fully reproduce the interactions of H_2_T4 with the complementary cluster of Asp/Glu residues at the h20S gate. This is also reflected by the fact that, unlike that previously obtained for the H_2_T4 [28], MTPyApi did not remain bound to the substrate gate in any of the other generated complexes (Figures S2 and S3). This finding reconciles with the kinetic data, which indicated a higher affinity of H_2_T4 for h20S as compared to MTPyApi.

Apart from these similarities, different binding modes were obtained according to the starting conformation of the apidaecin tail, and the best-docked solution (presenting the most favorable interaction energy: −143.941 kcal/mol; Tables S4 and S5) was achieved by starting with the apidaecin tail in the extended conformation interacting with the α5-α6 groove (Figure 7). This solution represents the best-docked complex both after the Monte Carlo docking procedure and after the molecular dynamics (SA) simulation.

Thus, in the best-docked h20S/MTPyApi complex, the porphyrin ring of MTPyApi is positioned at the substrate gate and the apidaecin moiety at the α5-α6 groove. The full list of the interacting residues, together with the related binding energies and their eventual involvement in interaction with RPs, are reported in Table S10. Interestingly, the apidaecin binding site partially overlaps with that of the experimentally determined structure of the Pro–Arg rich inhibitor peptide (RRR-PRPP-YLP) bound to the y20S α5-α6 groove [35]. However, in the latter, only the binding site of the last three residues of the PR inhibitor was defined by the electron density map.

When starting from the conformation of the apidaecin tail folded on the porphyrin ring (Dock I), the solution presenting the ligand positioned at the substrate gate represents by far the best-docked complex after the Monte Carlo docking procedure, while it became the second-best-docked complex after the molecular dynamics (SA) simulation (Figure S8A,B and Table S4). Thus, in this case, the Monte-Carlo-best-docked complex and the SA-best-docked complexes showed different binding sites. Assuming that the ligand binding with the apidaecin tail is involved in intramolecular interactions, this result suggests that MTPyApi first interacts on the 20S surface (at the substrate gate) by the porphyrin moiety; afterwards, it enters the gate with the attached apidaecin sliding toward the substrate channel(s), thus competing with the substrate for the access to the catalytic sites. Noteworthy, the best-docked complex after the SA calculations presented the apidaecin Pro14 residue close to β5 catalytic threonine (first β ring) and the porphyrin moiety close to β2 catalytic threonine (second β ring) (Figure S8C). The full list of the interacting residues, together with the related binding energies and their eventual involvement in interaction with RPs, are reported in Tables S11 and S12.

Taken together, the results of our simulations suggest that MTPyApi is still able to bind to the h20S substrate gate, although the presence of just three positively charged N-methyl substituents compared to the four of H_2_T4 does not allow the perfect ionic match observed for this latter. Moreover, the bulk apidaecin tail, according to the conformation approaching the h20S α-ring surface, could either be placed at the α5-α6 groove or else drive the ligand inside the catalytic chamber to interact directly with the catalytic β2 and β5 subunits. Thus, while H_2_T4 acts exclusively at the level of the substrate gate, the apidaecin tail of MTPyApi, depending on its conformation, seems to play a crucial role in addressing the binding of porphyrin to h20S.

#### 3.4.2. Docking of TMPC

**Figure 8:**
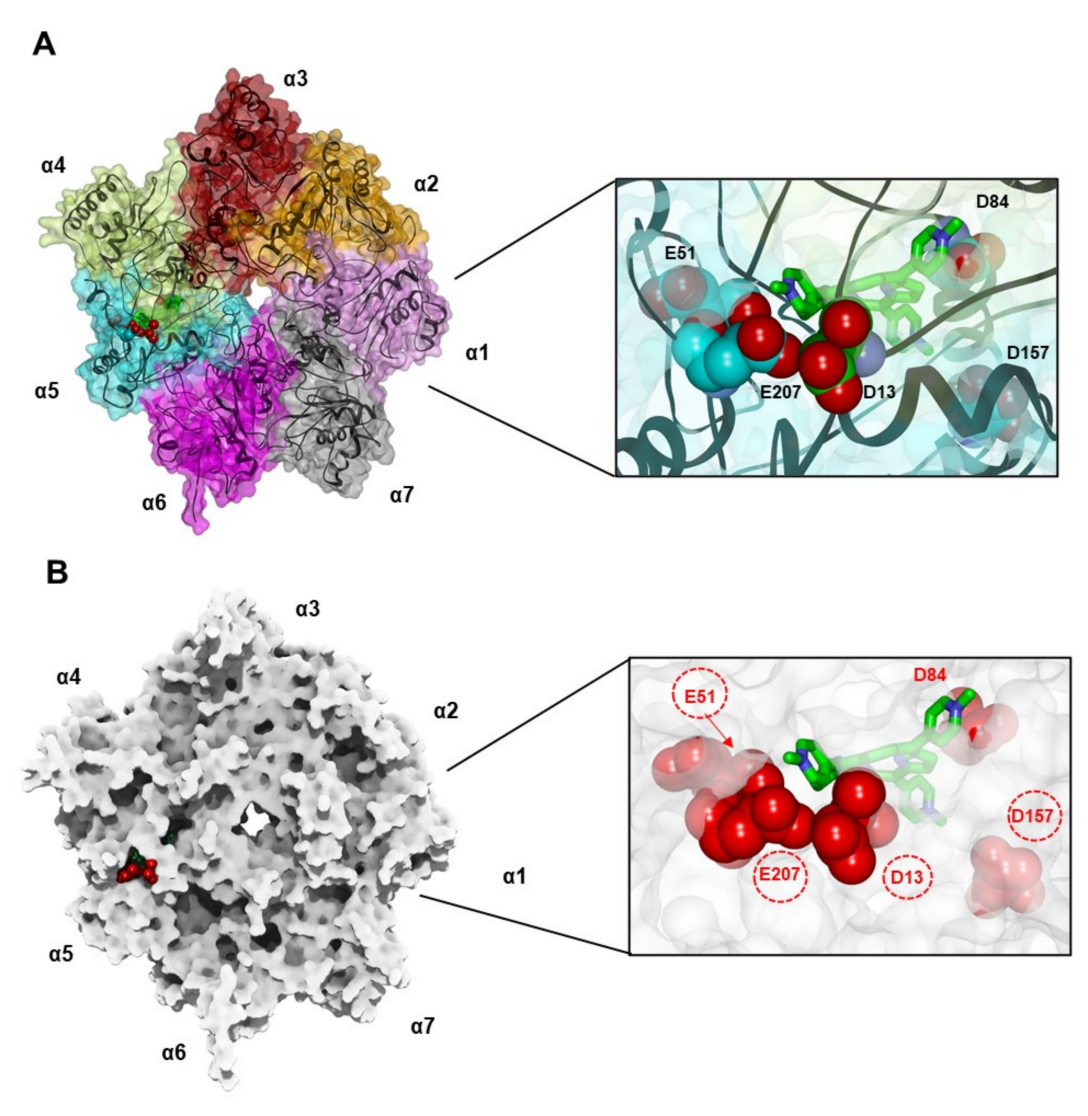
Selected docked complex of one TMPC molecule bound to human 20S (open conformation). (**A**,**B**): top and close-up view on the ligand binding site. The negative residues interacting with TMPC and those in common with Tris-T4 are displayed in CPK. (**A**) h20S backbone is displayed as black solid ribbons, while the transparent solvent accessible surface (SAS) and the displayed residues are colored in pink (α1), orange (α2), brown (α3), light green (α4), cyan (α5), magenta (α6), and gray (α7). TMPC is colored by atom type (C: black; N: blue; O: red), and its solvent accessibility surface is displayed (green). (**B**) h20S SAS is displayed as solid and colored in white. TMPC is colored by atom type (C: green; N: blue; O: red) and displayed in stick. The h20S interacting residues are colored in red. The negatively charged residues involved in ionic interactions with RPs are evidenced with a red dashed circle.

Regarding TMPC, we initially simulated the binding of the first molecule to both the closed and open forms of h20S. By comparing the Monte Carlo and SA interaction energy values of the best-docked complexes (Table S6 vs. S7), the TMPC molecule showed more favorable binding energies when docked to the open h20S structure than to the closed one. Since it is known that substrate-bound h20S shifts energetically toward the open state [21,50], this finding reconciles with kinetic data (Table 1), which indicated a higher affinity of TMPC for the substrate-bound form (*K_AL_*) than for the substrate-unbound form (*K_AU_*) of h20S. On the other hand, the comparison of the interaction energies of the best-docked solution of Tris-T4 and TMPC in complex with closed h20S, obtained at the end of the docking protocol (i.e., after the SA procedure), shows a slightly more favorable value for Tris-T4 [30] than for TMPC (Table S6 and Figure S4), in line with their observed K_AU_ values (Table 1). In addition, in agreement with the higher affinity of TMPC, as compared to Tris-T4, for the A_L_ form of h20S (Table 1), the interaction energies of the best-docked solution of TMPC in complex with open h20S (−75.61 kcal/mol; Table S7) are more favorable as compared to those previously calculated for Tris-T4 following the same docking procedure (−60.831 kcal/mol) [28]. Apart from the differences in the interaction energies, the results obtained when docking one molecule of TMPC and Tris-T4 to the open form of h20S showed, in both cases, the ligand bound to the α4-α5 groove interacting with key residues involved in h20S activation by RPs (Figure 8). The full list of the interacting residues, together with the related binding energies and their eventual involvement in interaction with RPs, are reported in Table S13.

**Figure 9:**
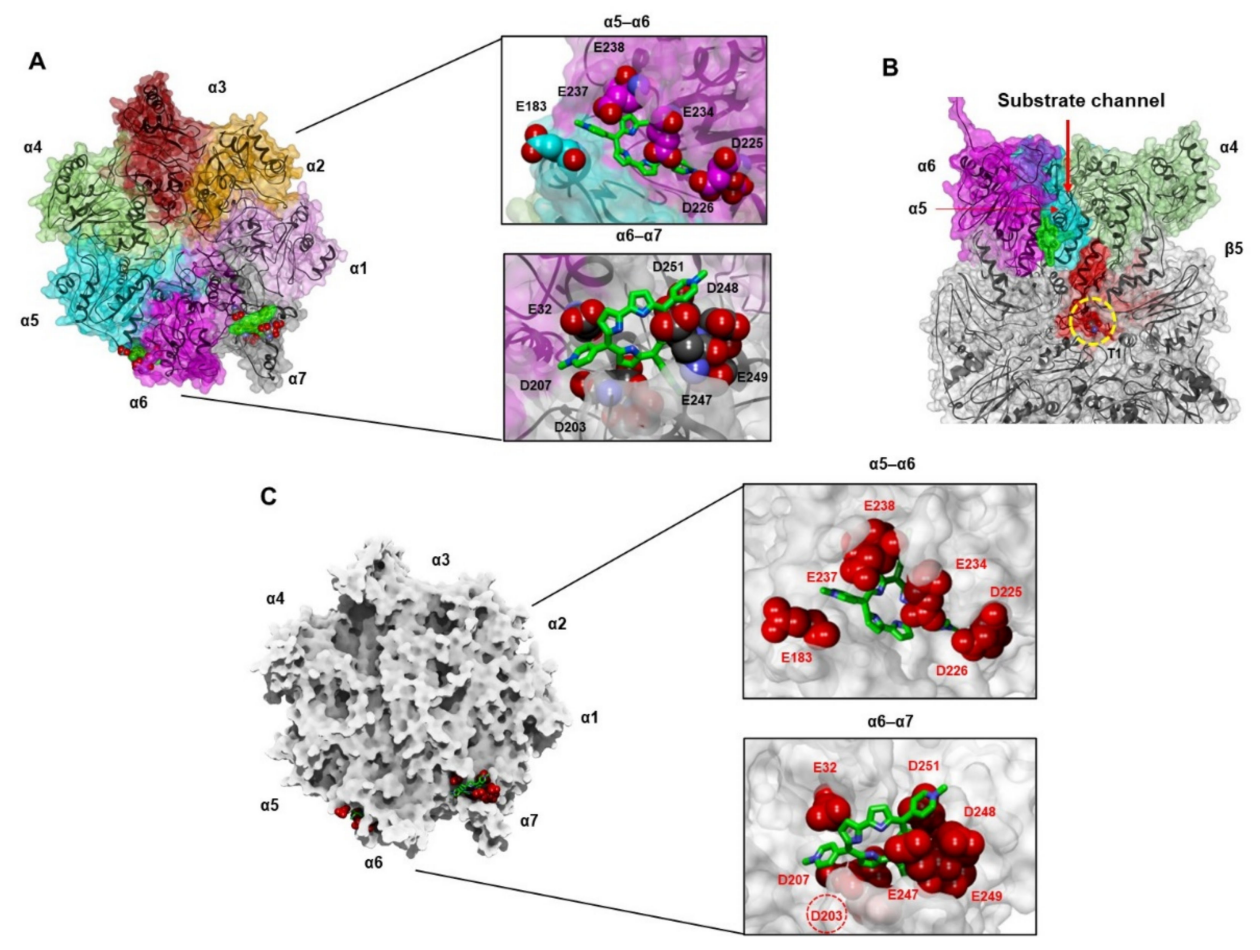
Selected docked complex of three TMPC molecules bound to human 20S (starting from the open conformation). (**A**,**C**): top and close-up view on the ligand binding sites at the α5-α6 and α6-α7 grooves. The negative residues interacting with TMPC and those in common with Tris-T4 are displayed in CPK. (**A**) h20S backbone is displayed as black solid ribbons, while the transparent solvent accessible surface (SAS) and the displayed residues are colored in pink (α1), orange (α2), brown (α3), light green (α4), cyan (α5), magenta (α6), and gray (α7). TMPC is colored by atom type (C: black; N: blue; O: red), and its SAS is displayed (green). (**C**) h20S SAS is displayed as solid and colored in white. TMPC is colored by atom type (C: green; N: blue; O: red) and displayed in stick. The h20S interacting residues are colored. The negative residues involved in ionic interactions with RPs are evidenced with a red dashed circle. (**B**) Transversal view and close-up view on the ligand binding site at α5-loop. For clarity of presentation, just a cross section of h20S proteasome is shown. h20S backbone is displayed as black solid ribbons, while the surface is colored in green (α4), cyan (α5), magenta (α6), violet (β2), and red (β5). TMPC is colored by atom type (C: black; N: blue; O: red), and its SAS is displayed (green).

Based on the outcome of the kinetic experiments, we continued our docking simulations considering the binding of two additional molecules of TMPC. When three molecules are bound to the surface of one of the α-ring of h20S proteasome, we can assume that the ligand-induced protein conformational changes drive the enzyme toward the B state (TMPC > 0.5 μM). The results obtained with the h20S closed structure were similar to those obtained for Tris-T4, with the ligand molecules still bound to the α-grooves (Table S14). However, it has to be underlined that, according to kinetic data, the amount of h20S not bound to the substrate (B_U_ form) is not relevant and the enzyme is entirely shifted to the B_L_ state (open h20S structure). Docking calculations performed with three TMPC molecules bound to the open h20S structure, which are thus representative of the B_U_ conformation, somewhat differ from those obtained with Tris-T4 (Figure 9 and Table S15). Indeed, the ligand molecule, initially bound to the α4-α5 groove, moves toward the center of the α-ring, enters in the substrate channel, and binds to the α5-loop and α6-loop at the substrate gate (Figure 9B). The α5-loop and the corresponding loop of the α6-subunit make part of the substrate access pore of h20S (α-annulus), regulating the translocation of substrates to the catalytic centers [8]; by consequence, the binding of TMPC to this region competes with the access of the substrate. It is very important to remark that, despite docking calculations having been performed using the open h20S conformation, the docked complex shows a closing of the substrate gate due to the moving of the N-terminal tail of the α3-subunit (Figure 9A). This is in agreement with the kinetic data indicating the presence of a competitive inhibitory binding site on h20S for TMPC, which becomes predominant from the functional standpoint (see Figure 4 and Figure 5) after the binding of three molecules of TMPC and which is not present in the case of Tris-T4. Moreover, according to the values reported in Table 1, this inhibitory site represents the TMPC highest affinity site on h20S, and this is mirrored by the values of the corresponding calculated interaction energies (Table S9).

The selected docking solutions were checked against the obtained NMR data (Table S16 and Figure S9). For this, we calculated the solvent accessible surface (SAS; H_2_O probe) of the TMPC molecules docked to the one or three binding sites, both starting from the closed and open h20S conformations. Therefore, we calculated the rate of SAS decrease for the corrole and N-methyl-pyridyl hydrogen atoms in our docking solutions with respect to their SAS in unbound TMPC (Table S16). As shown in Figure S9, in line with the NMR data (Figure 6), the hydrogen atoms of the directly bound pyrroles, together with those of adjacent methyl-pyridine rings of the TMPC molecules, resulted in the most shielding from the solvent when bound to the protein.

### 3.5. Porphyrins Inhibit h20S Proteasome in MCF7 Cells and Affect Cell Viability

**Figure 10:**
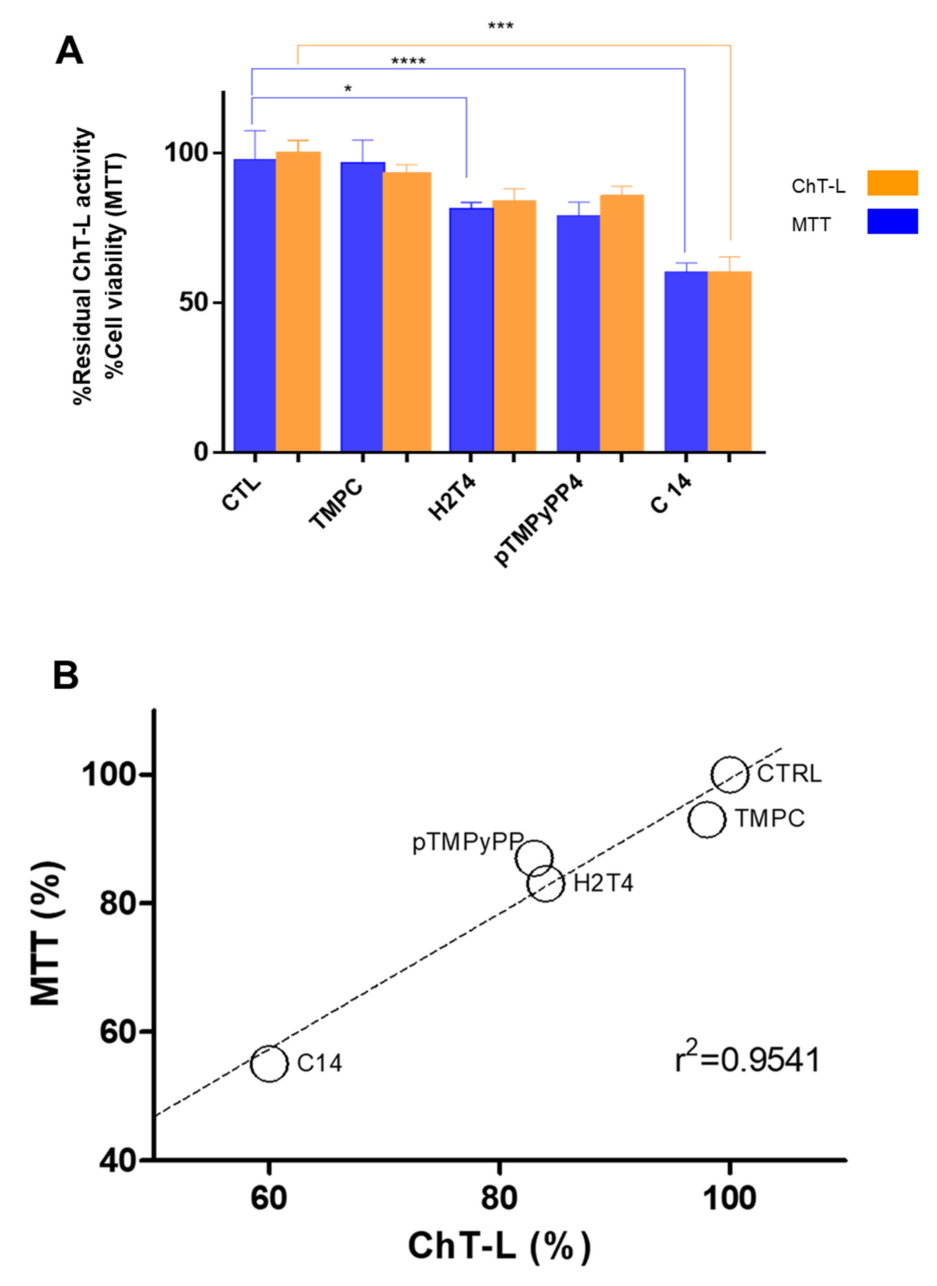
(**A**). Proteasome inhibition effects of different porphyrin derivatives (10 μM) monitored by luminescent probes (ProteasomeGlo) in living MCF7 cancer cells in the dark. Orange bars represent the residual ChT-L activity. Blue bars represent the cell viability of MCF7 cells treated with the same amount of inhibitor. Data from three independent experiments were normalized to untreated controls and are reported as mean ± standard deviation. Analysis of variance has been performed by one-way ANOVA followed by statistical Sidak’s test (* *p* < 0.05; *** *p* < 0.01; **** *p* < 0.0001). The relationship existing between proteasome inhibition and pro-apoptotic potential is supported by (**B**), which reports a linear fit of the residual ChT-L proteasome activity in living cells, plotted versus the cell viability detected by MTT.

Along with a mechanistic investigation on MTPyApi and TMPC, we performed a preliminary investigation in cell culture in order to establish whether the effect, detected in isolated proteasome samples, may be related to what occurs in the cell so as to foresee the potential of porphyrin- and corrole-based ligands as therapeutical tools. In particular, we focused on the relationship between proteasome inhibition and cytotoxic properties by evaluating the effect of some of these compounds on MCF7 breast cancer cell for 24 h in dark conditions. We included some representative porphyrin inhibitors, previously investigated (such as pTMPyPP4, H_2_T4, and its alkyl derivative, i.e., C14), as well as the corrole modulator TMPC, testing their effect at 10 µM, a concentration value likely representative of a possible therapeutical application. At this concentration, only H_2_T4 and C14 showed a significant cytotoxic effect, detected by MTT assay (see Figure 10A, blue bars). Furthermore, in parallel with their viability, we measured the ChT-L residual activity by “in cell assay”, focusing our attention on the ChT-L activity only, which is known to recapitulate all the essential functions of proteasome.

As expected, the trend observed reflects only partially the effect previously determined in a cell-free proteasome assay. Even though all the tested compounds show an inhibitory trend/behavior, only the C14 treatment results in the most significant one. Parallel MTT experiments (Figure 10A, orange bars) show a linear relationship between proteasome inhibition versus cell viability (see Figure 10B). These results indicate that porphyrins in dark conditions may exert cytotoxic effects in MCF7 cancer cells, and these may be mainly attributed to their ability to inhibit proteasome activity.

## 4. Conclusions

This investigation provides further information on the relevance of charge distribution in steering the interaction of porphyrin derivatives with h20S proteasome, at the same time putting in evidence other factors playing a relevant role in regulating their functional effect. Indeed, MTPyApi, TMPC, and Tris-T4 share the same number of positive charges on the periphery of the planar pyrrole core (i.e., the three N-methyl pyridine substituents); however, either the removal of the phenyl ring in Tris-T4, as well as in the corrole derivative TMPC, or the introduction at its para position of the charged and flexible apidaecin tail in MTPyApi brought about a different interaction with the target protein. MTPyApi turned out to be a competitive inhibitor, as is H_2_T4, even though the latter is characterized by a higher affinity (as characterized by a lower equilibrium inhibitory dissociation constant). This higher affinity of H_2_T4 is likely related to the planar and symmetric arrangement of positive charges, which allows the ionic match with the cluster of negatively charged residues at the proteasome substrate gate, facilitating the closure of the gate through the simultaneous interaction with multiple α subunits. On the other hand, the lack of a positively charged N-methyl substituent and the insertion of the apidaecin moiety in MTPyApi does not allow the same perfect ionic match observed for H_2_T4. In the best-docked h20S/MTPyApi complex, the bulky and positively charged apidaecin tail is placed in the α5-α6 groove, recalling the binding of Arg–Pro-rich peptides, thus suggesting future structural modifications to improve the binding affinity.

In the case of the tri-cationic Tris-T4 and TMPC, which can accommodate simultaneously on multiple α-ring grooves, their concerted interactions clearly exert an allosteric effect, which induces the shift of the whole h20S proteasome structure toward a catalytically more active conformational state. Noteworthy, at higher concentrations, TMPC acts as a competitive inhibitor, an effect not observed with Tris-T4, putting in evidence the role played by the unsubstituted phenyl ring of Tris-T4 in h20S binding.

Therefore, tri-cationic porphyrinoids are able to work as allosteric activators of the h20S proteasome, a function that seems forbidden to porphyrinoids with a higher cationic character, which can only function as competitive inhibitors. In this respect, it is conceivable that tri-cationic porphyrinoids have a less efficient anti-tumoral effect with respect to other porphyrinoids with higher cationic character since, in this case, proteasome inhibition is required.

## Figures and Tables

**Table 1:** Parameters for the comparison of catalytic parameters for Tris-T4 and TMPC porphyrins on h20S according to Equation (3a–c).

	h20S		h20S + Tris-T4	h20S + TMPC
*k_catA_* (s^−1^)	11.5 ± 1.3	*K_AU_* (M^−1)^	2.0 (±0.3) × 10^4^	1.35 (±0.31) × 10^4^
*k_catB_* (s^−1^)	0.32 ± 0.05	*K_BU_* (M^−1)^	8.2 (±0.3) × 10^7^	2.55 (±0.47) × 10^8^
*K_mA_* (M)	1.95 (±0.37) × 10^−3^	*K_AL_* (M^−1)^	1.2 (±0.3) × 10^4^	3.5 (±0.6) × 10^5^
*K_mB_* (M)	1.5 (±0.3) × 10^−4^	*K_BL_* (M^−1^)	1.35 (±0.28) × 10^8^	3.1 (±0.6) × 10^8^
*L_U_*	3.0 (±0.5) × 10^−6^ (3)	*K_AI_* (M^−1^)	-	1.0 (±0.2) × 10^6^
*L_L_*	3.9 (±0.6) × 10^−5^ (3)	*K_BI_* (M^−1^)	-	2.05 (±0.47) × 10^9^

## Data Availability

Not applicable.

## Supplementary Materials

The following supporting information can be downloaded at: https://www.mdpi.com/article/10.3390/biom12060741/s1. Table S1: pKa values and ionic forms of TMPC and MTPyApi calculated using the algorithm ACD/pKa GALAS (ACD/Percepta software, Advanced Chemistry Development, Inc., Toronto, ON, Canada, 2017). Table S2: Identified structurally conserved regions (SCRs) of the human 20S proteasome using the Structure Prediction and Sequence Analysis server PredictProtein (http://www.predictprotein.org/), accessed on 21 July 2021). Table S3: Summary of MolProbity results obtained for the best-docked complexes docked porphyrin/20S complexes. Table S4: Nonbonded interaction energies (kcal/mol) of the 20S-MTPyApi complexes obtained by Monte Carlo and SA calculations using as starting binding site the substrate gate of 20S in the closed conformation (starting from the folded conformation of the apidaecin). Table S5: Nonbonded interaction energies (kcal/mol) of the 20S-MTPyApi complexes obtained by Monte Carlo and SA calculations using as starting binding site the substrate gate of 20S in the closed conformation (starting from the extended conformation of the apidaecin). Table S6: Nonbonded interaction energies (kcal/mol) of the 20S-TMPC complexes obtained by Monte Carlo and SA calculations using as starting binding site the α5-α6 groove of 20S in the closed conformation. Table S7: Nonbonded interaction energies (kcal/mol) of the 20S-TMPC complexes obtained by Monte Carlo and SA calculations using as starting binding site the α4–α5 groove of 20S in the open conformation. Table S8: Nonbonded interaction energies (kcal/mol) of the 20S-TMPC complexes obtained by Monte Carlo and SA calculations using as starting binding sites the α1-α2, α4-α5, and α5-α6 grooves of 20S in the closed conformation. Table S9: Nonbonded interaction energies (kcal/mol) of the 20S-TMPC complexes obtained by Monte Carlo and SA calculations using as starting binding sites the α1–α2, α4–α5, and α5–α6 grooves of 20S in the open conformation. Table S10: Ligand–residue nonbonded interaction energies (kcal/mol) of h20S-MTPyApi_1 (starting from the extended conformation of the apidaecin). Table S11: Ligand–residue nonbonded interaction energies (kcal/mol) of h20S-MTPyApi_1 (starting from the folded conformation of the apidaecin). Table S12: Ligand–residue nonbonded interaction energies (kcal/mol) of h20S-MTPyApi_7 (starting from the folded conformation of the apidaecin). Table S13: Ligand–residue nonbonded interaction energies (kcal/mol) of h20S/TMPC_5 (open 20S; one ligand molecule). Table S14: Ligand–residue nonbonded interaction energies (kcal/mol) of the closed h20S/TMPC best-docked complex (three ligand molecules). Table S15: Ligand–residue nonbonded interaction energies (kcal/mol) of the 20S in complex with three molecules of TMPC obtained by Monte Carlo and SA calculations using as starting binding sites the α5–α6, α4–α5, and α1–α2 grooves of 20S in the open conformation. Table S16: Calculated rate of solvent accessible surface (SAS) decrease for the corrole and N-methyl-pyridyl hydrogen atoms of TMPC bound to h20S in the selected docking solutions. Figure S1: Superimposition between the calculated global minimum conformers of Tris-T4 and TMPC by fitting the pyridine nitrogen atoms. Figure S2: Superimposition by Cα atoms of all the MTPyApi/h20S complexes generated by dynamic docking calculations (starting from the folded conformation of the apidaecin) using as starting structure the closed conformation of the human 20S proteasome. Figure S3: Superimposition by Cα atoms of all the MTPyApi/h20S complexes generated by dynamic docking calculations (starting from the extended conformation of the apidaecin) using as starting structure the closed conformation of the human 20S proteasome. Figure S4: Dynamic docking results obtained for TMPC using as starting structure one molecule bound at α5-α6 groove of human 20S proteasome in the closed conformation Figure S5: Dynamic docking results obtained for TMPC using as starting structure one molecule bound at α4-α5 groove of human 20S proteasome in the open conformation. Figure S6: Dynamic docking results obtained for TMPC using as starting structure three molecules bound at α1-α2, α4-α5 and α5-α6 grooves of human 20S proteasome in the closed conformation. Figure S7: Dynamic docking results obtained for TMPC using as starting structure three molecules bound at α1-α2, α4-α5 and α5-α6 grooves of human 20S proteasome in the open conformation. Figure S8: Selected docked complexes of MTPyApi bound to human 20S (closed conformation) obtained starting from the folded conformation of the apidaecin. Figure S9: Solvent accessible surface (SAS) of TMPC molecules docked to the closed and open h20S conformation.
