# Supplementary material for: Modulation of the 20S Proteasome Activity by Porphyrin Derivatives Is Steered through Their Charge Distribution
Source: Biomolecules. 2022 May 24;12(6):741. doi: 10.3390/biom12060741 (PMC9220251; doi:10.3390/biom12060741)
Supplement: Supplementary file 1 [file biomolecules-12-00741-s001.zip › biomolecules-1715049-supplementary.pdf]

Experimental section.

Analysis of SCRs of human 20S proteasome. To identify SCRs, the human 20S proteasome sequences were analyzed using the Structure Prediction and Sequence Analysis server PredictProtein (http://www.predictprotein. org/). In α1 subunits, 6 α-helix and 10 β-sheet secondary structures were predicted to be highly (α1, aa23–33; α2, aa85–104; α3, aa111–128; α4, aa172–183; α5, aa191–207; α6, aa232–243; β1, aa13–16; β2, aa38–43; β3, aa48–53; β4, aa68–72; β5, aa76–80; β6, aa135-143; β7, aa150-154; β8, aa160-168; β9, aa215-222; β10, aa226-229). In α2 subunits, 6 α-helix and 10 β-sheet secondary structures were predicted to be highly conserved (α1, aa20–30; α2, aa81–100; α3, aa107–124; α4, aa167–178; α5, aa184–198; α6, aa223–231; β1, aa9–13; β2, aa34–39; β3, aa44–49; β4, aa66–68; β5, aa72–76; β6, aa131-139; β7, aa145-149; β8, aa155-163; β9, aa208-214; β10, aa219-220). In α3 subunits, 6 α-helix and 10 β-sheet secondary structures were predicted to be highly conserved (α1, aa18–29; α2, aa80–100; α3, aa107–124; α4, aa168–178; α5, aa186–200; α6, aa230–248; β1, aa10–12; β2, aa33–38; β3, aa43–48; β4, aa66–68; β5, aa72–76; β6, aa131-139; β7, aa146-150; β8, aa157-164; β9, aa211-217; β10, aa224-227). In α4 subunits, 6 α-helix and 10 β-sheet secondary structures were predicted to be highly conserved (α1, aa17–27; α2, aa78–97; α3, aa104–121; α4, aa165–176; α5, aa183–198; α6, aa222–243; β1, aa6–10; β2, aa31–36; β3, aa41–46; β4, aa62–65; β5, aa69–73; β6, aa128-136; β7, aa143-147; β8, aa154-161; β9, aa206-212; β10, aa217-219). In α5 subunits, 6 α-helix and 10 β-sheet secondary structures were predicted to be highly conserved (α1, aa22–32; α2, aa83–102; α3, aa109–120; α4, aa174–185; α5, aa191–206; α6, aa231–240; β1, aa11–15; β2, aa36–41; β3, aa46–51; β4, aa66–70; β5, aa74–78; β6, aa138-146; β7, aa152-156; β8, aa162-170; β9, aa215-221; β10, aa226-228). In α6 subunits, 6 α-helix and 10 β-sheet secondary structures were predicted to be highly conserved (α1, aa19–30; α2, aa79–98; α3, aa105–122; α4, aa165–176; α5, aa184–199; α6, aa226–236; β1, aa10–13; β2, aa35–39; β3, aa45–49; β4, aa62–66; β5, aa70–74; β6, aa129-137; β7, aa143-147; β8, aa154-161; β9, aa210-216; β10, aa221-223). In α7 subunits, 6 α-helix and 10 β-sheet secondary structures were predicted to be highly conserved (α1, aa22–32; α2, aa83–102; α3, aa109–124; α4, aa170–180; α5, aa187–202; α6, aa229–245; β1, aa13–15; β2, aa36–41; β3, aa46–51; β4, aa67–70; β5, aa74–78; β6, aa133-141; β7, aa148-152; β8, aa160-166; β9, aa212-219; β10, aa224-227). In β1 subunits, 5 α-helix and 11 β-sheet secondary structures were predicted to be highly conserved (α1, aa52–69; α2, aa76–90; α3, aa132-143; α4, aa149–166; α5, aa191–200; β1, aa2–8; β2, aa12–16; β3, aa25–27; β4, aa34–37; β5, aa41–45; β6, aa95-103; β7, aa110-114; β8, aa120-122; β9, aa124-128; β10, aa174-180; β11, aa185-189). In β2 subunits, 5 α-helix and 12 β-sheet secondary structures were predicted to be highly conserved (α1, aa52–67; α2, aa76–90; α3, aa131-142; α4, aa148–165; α5, aa194–201; β1, aa2–8; β2, aa12–17; β3, aa25–27; β4, aa33–37; β5, aa41–45; β6, aa95-103; β7, aa109-113; β8, aa123-127; β9, aa173-179; β10, aa184-186; β11, aa210-213; β12, aa215-225). In β3 subunits, 4 α-helix and 10 β-sheet secondary structures were predicted to be highly conserved (α1, aa56–76; α2, aa83–97; α3, aa142-152; α4, aa159–175; β1, aa9–14; β2, aa18–24; β3, aa32–34; β4, aa41–44; β5, aa48–52; β6, aa103-111; β7, aa119-123; β8, aa134-138; β9, aa184-190; β10, aa194-199). In β4 subunits, 5 α-helix and 10 β-sheet secondary structures were predicted to be highly conserved (α1, aa32–35; α2, aa50–70; α3, aa77–93; α4, aa136-147; α5, aa153–170; β1, aa3–8; β2, aa12–17; β3, aa26–27; β4, aa36–38; β5, aa42–46; β6, aa99-107; β7, aa114-118; β8, aa129-132; β9, aa178-184; β10, aa189-190). In β5 subunits, 6 α-helix and 11 β-sheet secondary structures were predicted to be highly conserved (α1, aa31–34; α2, aa49–69; α3, aa76–91; α4, aa132-143; α5, aa149–166; α6, aa189–200; β1, aa2–8; β2, aa12–16; β3, aa25–26; β4, aa35–37; β5, aa41–45; β6, aa96-104; β7, aa110-114; β8, aa120-122; β9, aa124-128; β10, aa174-180; β11, aa185-186). In β6 subunits, 4 α-helix and 11 β-sheet

secondary structures were predicted to be highly conserved (α1, aa58–77; α2, aa85–99; α3, aa142-152; α4, aa168–184; β1, aa5–6; β2, aa11–16; β3, aa20–26; β4, a34–36; β5, aa42–46; β6, aa50-54; β7, aa105-113; β8, aa120-124; β9, aa134-138; β10, aa193-199; β11, aa203-210). In β7 subunits, 6 α-helix and 10 β-sheet secondary structures were predicted to be highly conserved (α1, aa39–41; α2, aa57–77; α3, aa85-100; α4, aa143–154; α5, aa162–179; α6, aa207–216; β1, aa10–16; β2, aa20–25; β3, aa33–35; β4, aa42–45; β5, aa49–53; β6, aa107-115; β7, aa121-125; β8, aa136-139; β9, aa187-193; β10, aa198-199).

Table S1. Pka values and ionic forms of **TMPC** and **MTPyApi** calculated using the algorithm ACD/pKa GALAS (ACD/Percepta software, Advanced Chemistry Development, Inc., Toronto, ON, Canada, 2017).

Compound	pka1	pka2	Ionic forms at pH 7.2 (%)
**TMPC**	6.1±1.4	9.1±1.0	Tetra-cationic (7)<br>Tri-cationic (92)<br>Di-cationic (1)
**MTPyApi**[a]	6.4±0.8	11.4±1.1	Epta-cationic (20)<br>Esa-cationic (80)

[a] In the case of **MTPyApi** are reported only the pka values relative to the protonation states of porphyrin ring.

Table S2. Identified structurally conserved regions (SCRs) of the human 20S proteasome using the Structure Prediction and Sequence Analysis server PredictProtein (http://www.predictprotein. org/).(Accessed on 21 July 2021).

Subunits	Amino acid numbering	Secondary structure
α1	13-16	β-sheet
	23-33	α-helix
	38-43	β-sheet
	48-53	β-sheet
	68-72	β-sheet
	76-80	β-sheet
	85-104	α-helix
	111-128	α-helix
	135-143	β-sheet
	150-154	β-sheet
	160-168	β-sheet
	172-183	α-helix
	191-207	α-helix
	215-222	β-sheet
	226-229	β-sheet
	232-243	α-helix
α2	9-13	β-sheet
	20-30	α-helix
	34-39	β-sheet
	44-49	β-sheet
	66-68	β-sheet
	72-76	β-sheet
	81-100	α-helix
	107-124	α-helix
	131-139	β-sheet
	145-149	β-sheet
	155-163	β-sheet

	167-178	α-helix
	184-198	α-helix
	208-214	β-sheet
	219-220	β-sheet
	223-231	α-helix
α3	10-12	β-sheet
	18-29	α-helix
	33-38	β-sheet
	43-48	β-sheet
	66-68	β-sheet
	72-76	β-sheet
	80-100	α-helix
	107-124	α-helix
	131-139	β-sheet
	146-150	β-sheet
	157-164	β-sheet
	168-178	α-helix
	186-200	α-helix
	211-217	β-sheet
	224-227	β-sheet
	230-248	α-helix
α4	6-10	β-sheet
	17-27	α-helix
	31-36	β-sheet
	41-46	β-sheet
	62-65	β-sheet
	69-73	β-sheet
	78-97	α-helix
	104-121	α-helix
	128-136	β-sheet
	143-147	β-sheet
	154-161	β-sheet
	165-176	α-helix
	183-198	α-helix
	206-212	β-sheet
	217-219	β-sheet
	222-243	α-helix
α5	11-15	β-sheet
	22-32	α-helix
	36-41	β-sheet
	46-51	β-sheet
	66-70	β-sheet
	74-78	β-sheet
	83-102	α-helix
	109-120	α-helix
	138-146	β-sheet
	152-156	β-sheet
	162-170	β-sheet
	174-185	α-helix
	191-206	α-helix

	215-221	β-sheet
	226-228	β-sheet
	231-240	α-helix
α6	10-13	β-sheet
	19-30	α-helix
	35-39	β-sheet
	45-49	β-sheet
	62-66	β-sheet
	70-74	β-sheet
	79-98	α-helix
	105-122	α-helix
	129-137	β-sheet
	143-147	β-sheet
	154-161	β-sheet
	165-176	α-helix
	184-199	α-helix
	210-216	β-sheet
	221-223	β-sheet
	226-236	α-helix
α7	13-15	β-sheet
	22-32	α-helix
	36-41	β-sheet
	46-51	β-sheet
	67-70	β-sheet
	74-78	β-sheet
	83-102	α-helix
	109-124	α-helix
	133-141	β-sheet
	148-152	β-sheet
	160-166	β-sheet
	170-180	α-helix
	187-202	α-helix
	212-219	β-sheet
	224-227	β-sheet
	229-245	α-helix
β1	2-8	β-sheet
	12-16	β-sheet
	25-27	β-sheet
	34-37	β-sheet
	41-45	β-sheet
	52-69	α-helix
	76-90	α-helix
	95-103	β-sheet
	110-114	β-sheet
	120-122	β-sheet
	124-128	β-sheet
	132-143	α-helix
	149-166	α-helix
	174-180	β-sheet
	185-189	β-sheet

	191-200	α-helix
β2	2-8	β-sheet
	12-17	β-sheet
	25-27	β-sheet
	33-37	β-sheet
	41-45	β-sheet
	52-67	α-helix
	76-90	α-helix
	95-103	β-sheet
	109-113	β-sheet
	123-127	β-sheet
	131-142	α-helix
	148-165	α-helix
	173-179	β-sheet
	184-186	β-sheet
	194-201	α-helix
	210-213	β-sheet
	215-225	β-sheet
β3	9-14	β-sheet
	18-24	β-sheet
	32-34	β-sheet
	41-44	β-sheet
	48-52	β-sheet
	56-76	α-helix
	83-97	α-helix
	103-111	β-sheet
	119-123	β-sheet
	134-138	β-sheet
	142-152	α-helix
	159-175	α-helix
	184-190	β-sheet
	194-199	β-sheet
β4	3-8	β-sheet
	12-17	β-sheet
	26-27	β-sheet
	32-35	α-helix
	36-38	β-sheet
	42-46	β-sheet
	50-70	α-helix
	77-93	α-helix
	99-107	β-sheet
	114-118	β-sheet
	129-132	β-sheet
	136-147	α-helix
	153-170	α-helix
	178-184	β-sheet
	189-190	β-sheet
β5	2-8	β-sheet
	12-16	β-sheet
	25-26	β-sheet

	31-34	α-helix
	35-37	β-sheet
	41-45	β-sheet
	49-69	α-helix
	76-91	α-helix
	96-104	β-sheet
	110-114	β-sheet
	120-122	β-sheet
	124-128	β-sheet
	132-143	α-helix
	149-166	α-helix
	174-180	β-sheet
	185-186	β-sheet
	189-200	α-helix
β6	5-6	β-sheet
	11-16	β-sheet
	20-26	β-sheet
	34-36	β-sheet
	42-46	β-sheet
	50-54	β-sheet
	58-77	α-helix
	85-99	α-helix
	105-113	β-sheet
	120-124	β-sheet
	134-138	β-sheet
	142-152	α-helix
	168-184	α-helix
	193-199	β-sheet
	203-210	β-sheet
β7	10-16	β-sheet
	20-25	β-sheet
	33-35	β-sheet
	39-41	α-helix
	42-45	β-sheet
	49-53	β-sheet
	57-77	α-helix
	85-100	α-helix
	107-115	β-sheet
	121-125	β-sheet
	136-139	β-sheet
	143-154	α-helix
	162-179	α-helix
	187-193	β-sheet
	198-199	β-sheet
	207-216	α-helix

Table S3. Summary of Molprobity results obtained for the best-docked complexes docked porphyrin/20S complexes.

Structure	Residues favored regions	Residues allowed regions	Residues outliers	Poor rotamers
h20S/MTPyApi_1[a]	79.8%	17.1%	3.1%	1.8%
h20S/MTPyApi_7[a]	79.6%	17.5%	2.9%	2.0%
h20S/MTPyApi_1[b]	79.4%	17.5%	3.1%	2.0%
h20S/TMPC_2 (closed)	79.1%	17.7%	3.2%	1.9%
h20S/TMPC_5 (open)	77.7%	18.9%	3.4%	2.2%
h20S/TMPC_1 (closed)	79.5%	17.5%	3.0%	2.1%
h20S/TMPC_6 (open)	78.7%	17.9%	3.4%	2.2%

[a] Starting from the folded conformation of the apidaecin. [b] Starting from the extended conformation of the apidaecin.

Table S4. Nonbonded interaction energies (kcal/mol) of the 20S-**MTPyApi** complexes obtained by Monte Carlo and SA calculations using as starting binding site the substrate gate of 20S in the closed conformation (starting from the folded conformation of the apidaecin).

Cplx	Nonbonded interaction energies (kcal/mol)	
	Monte Carlo Simulation	Simulated Annealing Simulation
**1**[a]	-110.146	-134.065
**2**	-38.430	-115.038
**3**	-57.097	-91.686
**4**	-41.720	-73.111
**5**	-34.265	-115.723
**6**	-17.937	-62.663
**7**[a]	-37.560	-141.481
**8**	-57.349	-132.562

[a]Selected complexes.

Table S5. Nonbonded interaction energies (kcal/mol) of the 20S-**MTPyApi** complexes obtained by Monte Carlo and SA calculations using as starting binding site the substrate gate of 20S in the closed conformation (starting from the extended conformation of the apidaecin).

Cplx	Nonbonded interaction energies (kcal/mol)	
	Monte Carlo Simulation	Simulated Annealing Simulation
**1**[a]	-116.404	-143.941
**2**	-22.153	-64.411
**3**	-22.084	-41.168
**4**	-26.416	-142.841
**5**	-40.372	-134.006
**6**	-51.477	-74.004
**7**	-60.818	-90.001
**8**	-49.842	-60.840

[a]Selected complex.

Table S6. Nonbonded interaction energies (kcal/mol) of the 20S-**TMPC** complexes obtained by Monte Carlo and SA calculations using as starting binding site the α5-α6 groove of 20S in the closed conformation.

Cplx	Nonbonded interaction energies (kcal/mol)	
	Monte Carlo Simulation	Simulated Annealing Simulation
**1**	-34.73	-54.97
**2**[a]	-42.64	-55.10
**3**	-37.85	-39.29
**4**	-38.85	-49.90
**5**	-38.97	-50.23
**6**	-2.92	-25.30
**7**	-3.14	-40.70
**8**	-2.86	-31.26
**9**	-4.31	-25.12
**10**	-23.22	-73.59
**11**	-28.63	-44.29
**12**	-2.90	-13.93

[a]Selected complex.

Table S7. Nonbonded interaction energies (kcal/mol) of the 20S-**TMPC** complexes obtained by Monte Carlo and SA calculations using as starting binding site the α4-α5 groove of 20S in the open conformation.

Cplx	Nonbonded interaction energies (kcal/mol)	
	Monte Carlo Simulation	Simulated Annealing Simulation
**1**	-44.01	-17.97
**2**	-38.89	-57.74
**3**	-41.89	-55.37
**4**	-42.73	-47.17
**5**[a]	-56.11	-75.61
**6**	-3.62	-20.67
**7**	-18.84	-39.25
**8**	-26.72	-34.00
**9**	-25.78	-33.81
**10**	-23.36	-24.72
**11**	-36.65	-46.14
**12**	-5.31	-40.53

aSelected complex.

Table S8. Nonbonded interaction energies (kcal/mol) of the 20S-**TMPC** complexes obtained by Monte Carlo and SA calculations using as starting binding sites the α1-α2, α4-α5 and α5-α6 grooves of 20S in the closed conformation.

Cplx	Nonbonded interaction energies (kcal/mol)	
	Monte Carlo Simulation	Simulated Annealing Simulation
**1**[a]	-118.17	-151.91
**2**	-63.99	-129.05
**3**	-90.22	-137.65
**4**	-89.93	-105.36
**5**	-76.13	-145.32
**6**	-102.81	-98.08

**7**	-111.63	-132.32
**8**	-82.42	-112.12
**9**	-87.84	-119.15
**10**	-90.14	-149.75

[a]Selected complex.

Table S9. Nonbonded interaction energies (kcal/mol) of the 20S-**TMPC** complexes obtained by Monte Carlo and SA calculations using as starting binding sites the α1-α2, α4-α5 and α5-α6 grooves of 20S in the open conformation.

**Cplx**	**Nonbonded interaction energies (kcal/mol)**	
	Monte Carlo Simulation	Simulated Annealing Simulation
**1**	-117.21	-158.32
**2**	-36.11	-121.50
**3**	-73.81	-135.06
**4**	-118.01	-182.95
**5**	-85.34	-190.87
**6**[a]	-125.08	-215.59
**7**	-84.35	-149.68
**8**	-94.58	-182.63
**9**	-121.44	-168.12
**10**	-87.11	-137.78

[a]Selected complex.

Table S10. Ligand-residue nonbonded interaction energies (kcal/mol) of h20S-**MTPyApi**_1 (starting from the extended conformation of the apidaecin). The residues involved in the interaction with RPs are noted and the corresponding RPs are reported.

**h20S amino acids**	**Subunit**	**Nonbonded interaction Energy (kcal/mol)**	**RPs**[a]
G8	α1	-1.111	
F9	α1	-3.981	
D10	α1	-3.991	
H12	α1	-3.108	
I13	α1	-0.264	
R4	α3	-2.655	
Y5	α3	-2.552	
D6	α3	-3.312	
S7	α3	-2.998	
P12	α4	-0.823	
Y8	α5	-4.182	
D9[c]	α5	-1.562	PA200
R10	α5	-0.972	
V12	α5	-0.735	
T14	α5	-1.308	
S16	α5	-0356	
P17	α5	-0.847	
E18[c]	α5	-7.108	19S (Sa-c), PA28, PA200
G19	α5	-0.861	
R20[c]	α5	-5.885	PA28
L21	α5	-2.528	
F22	α5	-5.244	
E25[c]	α5	-2.930	19S (Sd), PA28, PA200

E175	α5	-4.756	
Q178	α5	-2.330	
S179	α5	-1.330	
Q182	α5	-4.422	
E183	α5	-5.989	
F2	α6	-1.238	
N4	α6	-3.620	
Q5	α6	-1.312	
Y6	α6	-4.057	
D7	α6	-6.595	
N8	α6	-3.452	
D9	α6	-7.055	
V10	α6	-0.223	
T11	α6	-1.106	
V12	α6	-0.195	
W13	α6	-0.139	
Y24	α6	-1.674	
E27[c]	α6	-0.295	PA200
Q31	α6	-0.086	
G32[b]	α6	-0.038	19S (Sc-d)
Q53	α6	-5.957	
S54	α6	-3.298	
E55	α6	-9.281	
L56	α6	-0.238	
A57	α6	-0.180	
A58	α6	-0.059	
H59	α6	-0.062	
E202[c]	α6	-3.558	PA200
Q203	α6	-0.183	
K208	α6	-0.055	
G5	α7	-0.182	
T6	α7	-0.962	
G7	α7	-2.538	
Y8	α7	-0.777	
D9[c]	α7	-3.421	19S (Sd)
L10	α7	-1.792	
S11	α7	-0.328	

[a]19S functionals states involved in the reported interaction are specified in brackets. [b]Amino acids of the C-terminal tail of Rpt5 (aa426-aa439; α5/α6) (19S) and PA200 (aa1830-aa1843) having at least one atom within a 4 Å radius from any given h20S residue. [c]Negatively and positively charged residues involved in ionic interaction with RPs (i.e., PA28, PA200 and 19S).

Table S11. Ligand-residue nonbonded interaction energies (kcal/mol) of h20S-**MTPyApi_**1 (starting from the folded conformation of the apidaecin). The residues involved in the interaction with RPs are noted and the corresponding RPs are reported.

h20S amino acids	Subunit	Nonbonded interaction Energy (kcal/mol)	RPs[a]
R3	α1	-4.293	
G4	α1	-0.793	
S5	α1	-2.967	
S6	α1	-3.879	

A7	$\alpha1$	-4.337	
G8	$\alpha1$	-3.250	
F9	$\alpha1$	-6.924	
D10	$\alpha1$	-7.039	
I13	$\alpha1$	-0.450	
I15	$\alpha1$	-0.203	
G158	$\alpha1$	-0.029	
G169	$\alpha1$	-0.013	
S9	$\alpha2$	-0.665	
L10	$\alpha2$	-3.816	
T11	$\alpha2$	-2.579	
T12	$\alpha2$	-2.676	
F13	$\alpha2$	-3.341	
S14	$\alpha2$	-2.862	
P15	$\alpha2$	-0.880	
S16	$\alpha2$	-0.809	
G17	$\alpha2$	-0.212	
K18	$\alpha2$	-0.836	
L19	$\alpha2$	-0.678	
V20	$\alpha2$	-2.412	
E23[c]	$\alpha2$	-1.719	PA28
G31[b]	$\alpha2$	-0.025	19S (Sd)
Q123	$\alpha2$	-0.093	
G126	$\alpha2$	-0.055	
G131	$\alpha2$	-0.021	
G153	$\alpha2$	-0.032	
G164	$\alpha2$	-0.013	
S2	$\alpha3$	-0.734	
R3	$\alpha3$	-0.497	
R4	$\alpha3$	-1.468	
Y5	$\alpha3$	-4.148	
D6	$\alpha3$	-11.515	
S7	$\alpha3$	-0.593	
T9	$\alpha3$	-3.472	
T10	$\alpha3$	-0.894	
I11	$\alpha3$	-2.222	
G16	$\alpha3$	-0.013	
Y19	$\alpha3$	-1.302	
Y23	$\alpha3$	-0.991	
I28	$\alpha3$	-0.272	
G29	$\alpha3$	-0.011	
G32	$\alpha3$	-0.015	
G125	$\alpha3$	-0.139	
G14	$\alpha4$	-0.025	
G123	$\alpha4$	-0.054	
S6	$\alpha5$	-0.637	
Y8	$\alpha5$	-7.281	
D9[c]	$\alpha5$	-7.153	PA200
G11	$\alpha5$	-0.296	
N13	$\alpha5$	-0.355	
T14	$\alpha5$	-0.515	

F22	α5	-0.275
G124	α5	-0.026
G131	α5	-0.036
G160	α5	-0.012
Q5	α6	-3.020
Y6	α6	-1.458
D7	α6	-8.844
N8	α6	-2.462
D9	α6	-8.759
V12	α6	-0.042
G113	α6	-0.010
G5	α7	-0.019
T6	α7	-0.854
G7	α7	-0.734
D9	α7	-4.821
P17	α7	-0.660
G19	α7	-0.034
G167	α7	-0.010

[a]19S functionals states involved in the reported interaction are specified in brackets. [b]Amino acids of the C-terminal tail of Rpt5 (aa426-aa439; α5/α6) (19S) and PA200 (aa1830-aa1843) having at least one atom within a 4 Å radius from any given h20S residue. [c]Negatively and positively charged residues involved in ionic interaction with RPs (i.e., PA28, PA200 and 19S).

Table S12. Ligand-residue nonbonded interaction energies (kcal/mol) of h20S-**MTPyApi**_7 (starting from the folded conformation of the apidaecin).

h20S amino acids	Subunit	Ring	Nonbonded interaction Energy (kcal/mol)
N24	β4	I	-0.242
I25	β4	I	-0.979
T21	β5	I	-2.582
A22	β5	I	-0.969
G23	β5	I	-2.931
A24	β5	I	-3.151
Y25	β5	I	-0.317
I26	β5	I	-1.072
A46	β5	I	-2.149
G47	β5	I	-1.820
G48	β5	I	-1.539
A49	β5	I	-0.413
A50	β5	I	-0.468
D51	β5	I	-2.952
C52	β5	I	-0.445
G94	β5	I	-0.747
L95	β5	I	-2.836
S96	β5	I	-1.947
M97	β5	I	-3.221
G98	β5	I	-1.202
T99	β5	I	-0.318
M100	β5	I	-0.146
D115	β5	I	-2.746
S116	β5	I	-1.231

E117	β5	I	-5.165
G118	β5	I	-0.075
G129	β5	I	-1.058
S130	β5	I	-3.355
G131	β5	I	-0.233
A168	β5	I	-0.496
Y169	β5	I	-4.915
G171	β5	I	-0.017
P4	β6	I	-0.102
Y5	β6	I	-2.039
V6	β6	I	-2.562
F7	β6	I	-3.571
N8	β6	I	-1.548
G9	β6	I	-0.172
G10	β6	I	-0.074
G32	β6	I	-0.278
F33	β6	I	-0.476
F101	β6	I	-0.420
F102	β6	I	-5.281
P103	β6	I	-4.009
Y104	β6	I	-1.273
Y105	β6	I	-4.683
P126	β6	I	-0.664
V127	β6	I	-1.391
G128	β6	I	-0.100
S140	β6	I	-0.282
Y188	β6	I	-0.158
Y141	β7	I	-0.410
G23	β1	II	-2.428
S24	β1	II	-3.553
Y25	β1	II	-1.295
I26	β1	II	-0.410
D51	β1	II	-2.893
F88	β2	II	-2.350
Q91	β2	II	-2.956
G92	β2	II	-2.172
Y93	β2	II	-3.861
I94	β2	II	-1.831
G95	β2	II	-0.281
P115	β2	II	-0.358
H116	β2	II	-5.558
G117	β2	II	-0.226
M127	β2	II	-0.067
I2	β3	II	-0.620
M3	β3	II	-0.559
S4	β3	II	-2.369
Y5	β3	II	-4.380
G7	β3	II	-0.088
G8	β3	II	-0.056
Q30	β3	II	-1.651
A31	β3	II	-0.231
L94	β3	II	-0.415

Y95	β3	II	-1.661
E96	β3	II	-1.923
F99	β3	II	-3.607
G100	β3	II	-0.270
P101	β3	II	-0.171
Y103	β3	II	-0.582
I126	β3	II	-2.126
G127	β3	II	-0.159
D177	β3	II	-0.743
V179	β3	II	-0.304
S180	β3	II	-0.107
G181	β3	II	-0.021
Y204H (water molecule)	β6 (bound to W403H)	I	-1.275
W403H (water molecule)	β6 (bound to Y5 (C=O))	I	-0.805
X309H (water molecule)	β5 bound to A22 (C=O)	I	-1.410

Table S13. Ligand-residue nonbonded interaction energies (kcal/mol) of h20S/**TMPC**_5 (open 20S; one ligand molecule). The residues involved in the interaction with RPs are noted and the corresponding RPs are reported.

h20S amino acids	Subunit	Nonbonded interaction Energy (kcal/mol)	RPs[a]
V9	α4	-1.747	
F10	α4	-2.552	
S11	α4	-2.092	
D13[c]	α4	-3.485	PA28
G14	α4	-2.617	
H15	α4	-4.418	
L16	α4	-4.485	
F17	α4	-1.866	
Q18	α4	-0.384	
G37	α4	-0.011	
G135	α4	-0.011	
S150[b]	α4	-0.329	19S (Sd)
G151	α4	-0.080	
T152	α4	-2.909	
G162	α4	-0.015	
A30	α5	-0.621	
I31	α5	-2.209	
G34	α5	-1.524	
S35[b]	α5	-4.828	19S (Sd)
T36	α5	-3.007	
A37	α5	-0.289	
G39	α5	-0.030	
V50	α5	-0.406	
E51	α5	-2.786	19S (Sa; Sb)
K52	α5	-1.897	

R53[b,c]	$\alpha5$	-0.014	19S (Sd); PA28; PA200
I54	$\alpha5$	-0.434	
S63	$\alpha5$	-0.829	
I64	$\alpha5$	-0.240	
S79	$\alpha5$	-2.537	
G80	$\alpha5$	-1.887	
L81[b]	$\alpha5$	-1.288	19S (Sd)
D84	$\alpha5$	-2.318	
G131	$\alpha5$	-0.011	
G138	$\alpha5$	-0.670	
V139	$\alpha5$	-0.635	
A140	$\alpha5$	-0.794	
G145	$\alpha5$	-0.012	
M156	$\alpha5$	-0.268	
D157[b]	$\alpha5$	-2.817	19S (Sa)
G171	$\alpha5$	-0.251	
E207[c]	$\alpha5$	-7.693	19S (Sa-d)
N211	$\alpha5$	-0.131	

[a]19S functional states involved in the reported interaction are specified in brackets. [b]Amino acids of the C-terminal tail of Rpt1 (aa421-aa433; $\alpha4/\alpha5$) (19S) having at least one atom within a 4 Å radius from any given h20S residue. [c]Negatively and positively charged residues involved in ionic interaction with RPs (i.e., PA28, PA200 and 19S).

Table S14. Ligand-residue nonbonded interaction energies (kcal/mol) of the closed h20S/**TMPC** best-docked complex (three ligand molecules). The residues involved in the interaction with RPs are noted and the RPs are reported.

Molecule	h20S amino acids	Subunit	Nonbondedinteraction Energy (kcal/mol)	RPs[a]
**TMPC_1 ($\alpha1$-$\alpha2$)**	E19[c]	$\alpha1$	-4.359	PA28
	G20	$\alpha1$	-1.926	
	L22[b]	$\alpha1$	-4.832	19S (Sa-Sd)
	Y23	$\alpha1$	-2.968	
	V25[b]	$\alpha1$	-1.186	19S (Sa; Sb)
	E26[c]	$\alpha1$	-9.131	19S (Sa-Sd); PA28
	F29[b]	$\alpha1$	-1.187	19S (Sa; Sb; Sc)
	G35	$\alpha1$	-0.013	
	G44	$\alpha1$	-0.022	
	C137	$\alpha1$	-0.043	
	G142	$\alpha1$	-0.010	
	D155[c]	$\alpha1$	-6.533	19S (Sd)
	G158	$\alpha1$	-0.062	
	Y159[b]	$\alpha1$	-2.298	19S (Sa; Sc; Sd)
	G162	$\alpha1$	-0.026	
	A167	$\alpha1$	-1.057	
	A168	$\alpha1$	-0.235	
	G169	$\alpha1$	-0.152	
	Q172	$\alpha1$	-2.619	
	T176	$\alpha1$	-0.368	
	A27	$\alpha2$	-1.654	
	A28[b]	$\alpha2$	-1.438	19S (Sd)

	V29	α2	-0.394	
	G31[b]	α2	-0.995	19S (Sd)
	G32[b]	α2	-0.040	19S (Sc)
	A33[b]	α2	-0.172	19S (Sa; Sc; Sd)
	G37	α2	-0.011	
	E49[c]	α2	-6.874	19S (Sa; Sc)
	L56	α2	-0.342	
	Y57	α2	-1.586	
	Y76	α2	-0.269	
	S77	α2	-0.261	
	G78[b]	α2	-2.076	19S (Sb)
	M79[b]	α2	-2.690	19S (Sa-Sd)
	G80[b]	α2	-0.850	19S (Sa)
	P81[b]	α2	-0.379	19S (Sc; Sd)
	G126	α2	-0.019	
	G131	α2	-0.072	
	G164	α2	-0.166	
**TMPC_2**	P12	α4	-0.762	
**(α5-α6)**	D13[c]	α4	-1.927	PA28
	G14	α4	-0.021	
	G151	α4	-0.013	
	G19[b]	α5	-1.645	19S (Sa; Sc); PA200
	L21[b]	α5	-0.519	19S (Sd)
	E25[b,c]	α5	-10.676	19S (Sc; Sd); PA28; PA200
	Y26	α5	-1.321	
	I28[b]	α5	-2.825	19S (Sa; Sc)
	E29[c]	α5	-7.475	19S (Sa)
	A30	α5	-0.178	
	L33[b]	α5	-1.336	19S (Sd)
	G34	α5	-0.100	
	T36	α5	-0.081	
	I38	α5	-0.203	
	G39	α5	-0.050	
	E51[c]	α5	-3.770	19S (Sa; Sb)
	G75	α5	-0.012	
	I170	α5	-3.138	
	G171	α5	-1.349	
	S172	α5	-4.274	
	A173	α5	-0.352	
	V205	α5	-0.655	
	M206	α5	-0.366	
	Q31	α6	-0.598	
	G32[b]	α6	-0.164	19S (Sc; Sd)
	Q53	α6	-2.243	
	S54	α6	-0.499	
	E55	α6	-2.389	
	L77[b]	α6	-1.005	19S (Sa-Sd); PA200
**TMPC_3**	P14	α3	-1.499	
**(α4-α5)**	E15[c]	α3	-2.083	19S (Sa; Sb; Sc); PA28
	G16	α3	-0.117	
	D13[c]	α4	-5.585	PA28

G14	α4	-2.210	
H15	α4	-3.151	
L16	α4	-2.045	
V19	α4	-2.122	
E20[c]	α4	-13.504	PA28
Y21	α4	-1.473	
Q23	α4	-2.013	
E24[c]	α4	-7.462	19S (Sa; Sb; Sd)
G151	α4	-0.075	
T152	α4	-1.120	
G162	α4	-0.134	
G164	α4	-0.139	
G202	α4	-0.017	
G203	α4	-0.013	
A30	α5	-0.541	
I31	α5	-1.522	
S35[b]	α5	-0.924	19S (Sd)
L81[b]	α5	-0.426	19S (Sd)

[a]19S functional states involved in the reported interaction are specified in brackets. [b]Amino acids of the C-terminal tail of Rpt5 (aa426-aa439; α5/α6), Rpt1 (aa421-433; α4/α5), Rpt3 (aa407-418; α1/α2) (19S) and PA200 (aa1830-aa1843) having at least one atom within a 4 Å radius from any given h20S residue. [c]Negatively and positively charged residues involved in ionic interaction with RPs (i.e., PA28, PA200 and 19S).

Table S15. Ligand-residue nonbonded interaction energies (kcal/mol) of the 20S in complex with three molecules of **TMPC** obtained by Monte Carlo and SA calculations using as starting binding sites the α5-α6, α4-α5 and α1-α2 grooves of 20S in the open conformation. The residues involved in the interaction with RPs are noted and the RPs are reported.

**Molecule**	**h20S amino acids**	**Subunit**	**Nonbondedinteraction Energy (kcal/mol)**	**RPs[a]**
**TMPC_1**	A92	α5	-0.129	
**(α5-loop α-annulus)**	E95	α5	-5.926	
	L119	α5	-2.494	
	A120	α5	-0.356	
	L121	α5	-0.346	
	Q122	α5	-0.214	
	F123	α5	-0.128	
	G124	α5	-0.065	
	E125	α5	-5.698	
	E126	α5	-8.033	
	D127	α5	-8.560	
	A128	α5	-1.842	
	G131	α5	-0.031	
	G138	α5	-0.021	
	G160	α5	-0.046	
	G71	α6	-0.021	
	L83	α6	-1.660	
	L84	α6	-0.556	
	N86	α6	-3.613	

	F87	α6	-5.612	
	Q90	α6	-4.520	
	E91	α6	-4.320	
	L93	α6	-0.553	
	D94	α6	-7.586	
	G113	α6	-0.038	
	G124	α6	-0.026	
	Y128	α6	-0.189	
	G129	α6	-0.030	
	G131	α6	-0.033	
**TMPC_2 (α6-α7)**	Q16	α6	-0.400	
	Q166	α6	-0.021	
	E32	α7	-7.034	
	N33	α7	-0.212	
	G39	α7	-0.015	
	G18	α7	-0.040	
	G160	α7	-0.018	
	Y199	α7	-0.304	
	I200	α7	-1.376	
	V201	α7	-1.299	
	D203[c]	α7	-6.181	19S (Sb)
	K206[c]	α7	-1.006	19S (Sa; Sb); PA28
	E207	α7	-9.729	
	L244	α7	-1.804	
	E246	α7	-3.865	
	E247	α7	-8.066	
	D248	α7	-5.494	
	E249	α7	-8.235	
	S250	α7	-2.979	
	D251	α7	-9.576	
	D252	α7	-2.909	
	N254	α7	-8.401	
	M255	α7	-5.416	
**TMPC_3 (α5-α6)**	G176	α5	-0.066	
	L181	α5	-0.662	
	Q182	α5	-3.618	
	E183	α5	-4.058	
	Y185	α5	-4.397	
	G32[b]	α6	-0.035	19S (Sc)
	L56	α6	-0.266	
	G162	α6	-0.016	
	G191	α6	-0.017	
	L192	α6	-0.698	
	T206	α6	-1.731	
	T207	α6	-3.563	
	K208	α6	-2.905	
	N209	α6	-0.818	
	V210	α6	-2.352	
	S211	α6	-0.384	
	G213	α6	-0.018	
	Y224	α6	-0.156	

D225	α6	-2.499
D226	α6	-6.998
V229	α6	-1.149
S230	α6	-0.790
F232	α6	-0.343
L233	α6	-3.411
E234	α6	-10.943
G235	α6	-0.206
L236	α6	-1.262
E237	α6	-9.820
E238	α6	-9.754
P240	α6	-0.083

[a]19S functional states involved in the reported interaction are specified in brackets. [b]Amino acids of the C-terminal tail of Rpt5 (aa426-aa439; α5/α6), Rpt1 (aa421-433; α4/α5), Rpt3 (aa407-418; α1/α2) (19S) and PA200 (aa1830-aa1843) having at least one atom within a 4 Å radius from any given h20S residue. [c]Negatively and positively charged residues involved in ionic interaction with RPs (i.e., PA28, PA200 and 19S).

Table S16. Calculated rate of solvent accessible surface (SAS) decrease for the corrole and N-methyl-pyridyl hydrogen atoms of **TMPC** bound to h20S in the selected docking solutions.

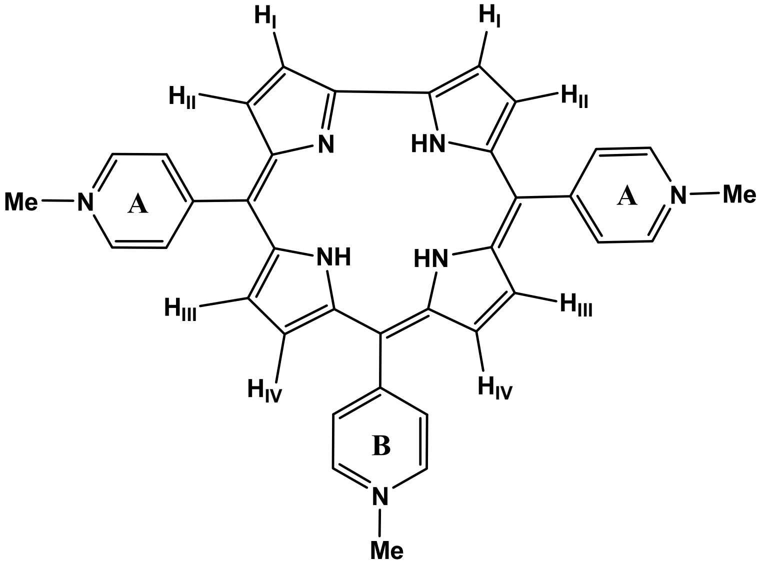

SAS decrease (%)

	Closed (1mol)	Open (1mol)	Closed (3mol)				Open (3 mol)			
	α5-α6	α4-α5	α1-α2	α4-α5	α5-α6	Average value	α5-α6	α6-α7	α5 loop	Average value
HH_{I} (Corrole)	61	72	47	50	65	54	0	0	0	0
H_{II} (Corrole)	20	52	74	82	38	65	0	0	0	0
H_{III} (Corrole)	32	54	0	8	0	3	100	76	0	59

H_{IV} (Corrole)	0	44	0	0	10	3	100	100	11	70
meta N-Me-Pyr (A)	24	67	87	9	36	44	0	0	0	0
ortho N-Me-Pyr (A)	73	100	36	32	28	32	27	28	0	18
meta N-Me-Pyr (B)	12	0	20	0	7	9	100	100	41	80
ortho N-Me-Pyr (B)	0	76	31	11	34	25	100	56	23	60

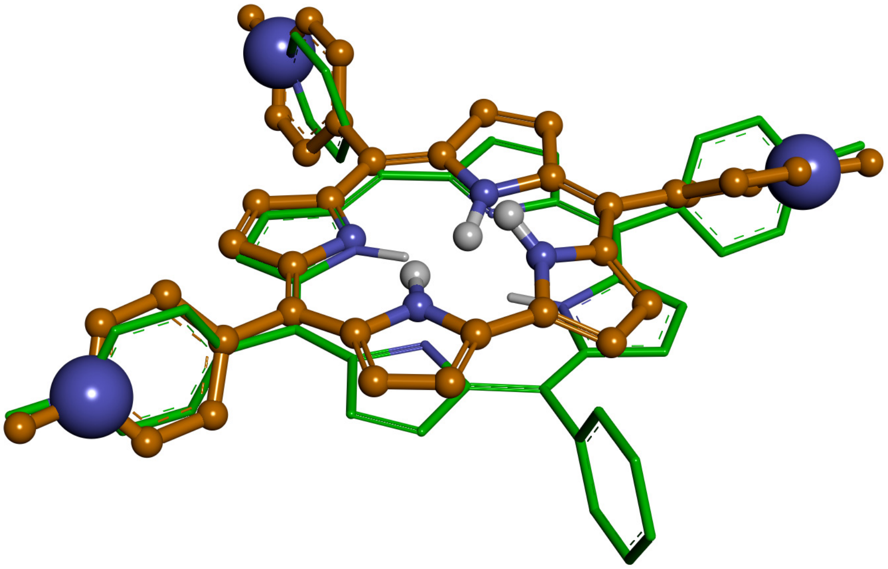

Figure S1. Superimposition between the calculated global minimum conformers of **Tris-T4** (displayed in stick; carbon atoms = green) and **TMPC** (displayed in ball&stick; carbon atoms = orange) by fitting the pyridine nitrogen atoms. Porphyrins are displayed with the pyridine nitrogen atoms evidenced in CPK (scaled by 50%) and colored by atom type. For sake of clarity, only the hydrogen atoms of the pyrrole nitrogen are displayed.

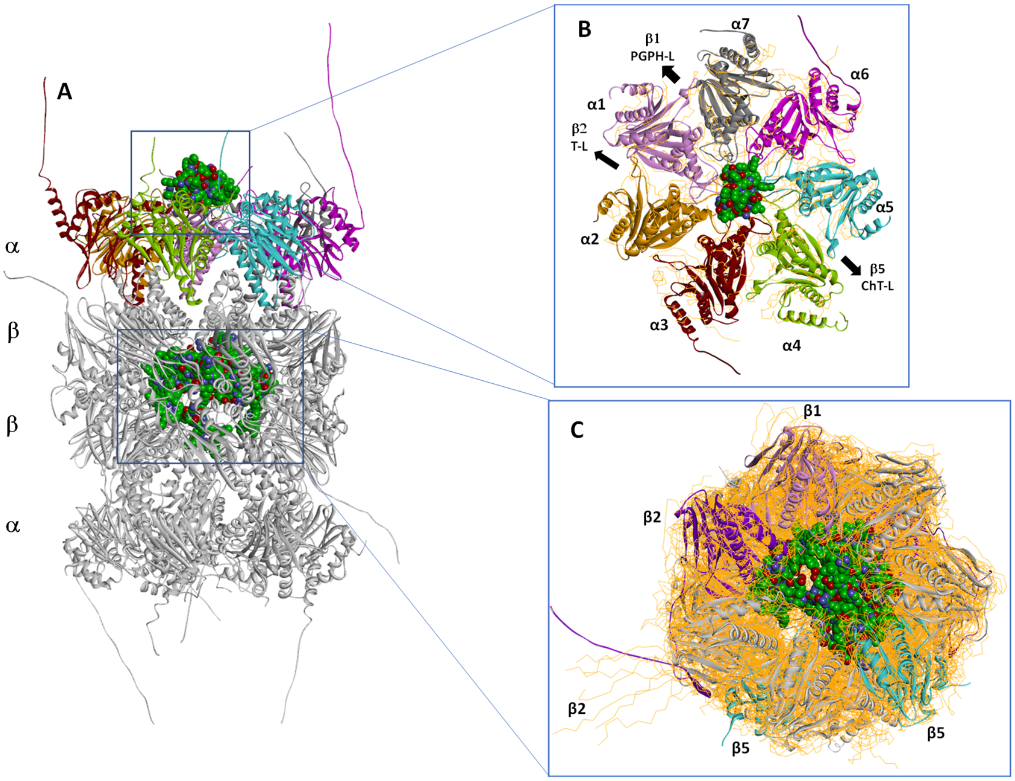

Figure S2. Superimposition by Cα atoms of all the **MTPyApi**/h20S complexes generated by dynamic docking calculations (starting from the folded conformation of the apidaecin) using as starting structure the closed conformation of the human 20S proteasome. A) Longitudinal view; the backbone of the starting complex is displayed as solid ribbons and colored in pink (α1), orange (α2), brown (α3), light green (α4), cyan (α5), magenta (α6), and gray (α7, α subunits of the second ring and all β subunits); **MTPyApi** is displayed as CPK and colored by atom type (C = green); B) Top view of dynamic docking results without the two rings of β subunits and the second ring of α subunits. C) Top view of dynamic docking results without the first ring of α subunits. The backbone of the calculated complexes is displayed as line ribbons and colored in orange (B and C). The porphyrin ligands are colored by atom type (C: green N: blue; O: red) and displayed as CPK. In B the α subunits and the catalytic β subunits are labeled. In C the catalytic β subunits are labeled and colored in pink (β1), violet (β2), and cyan (β5).

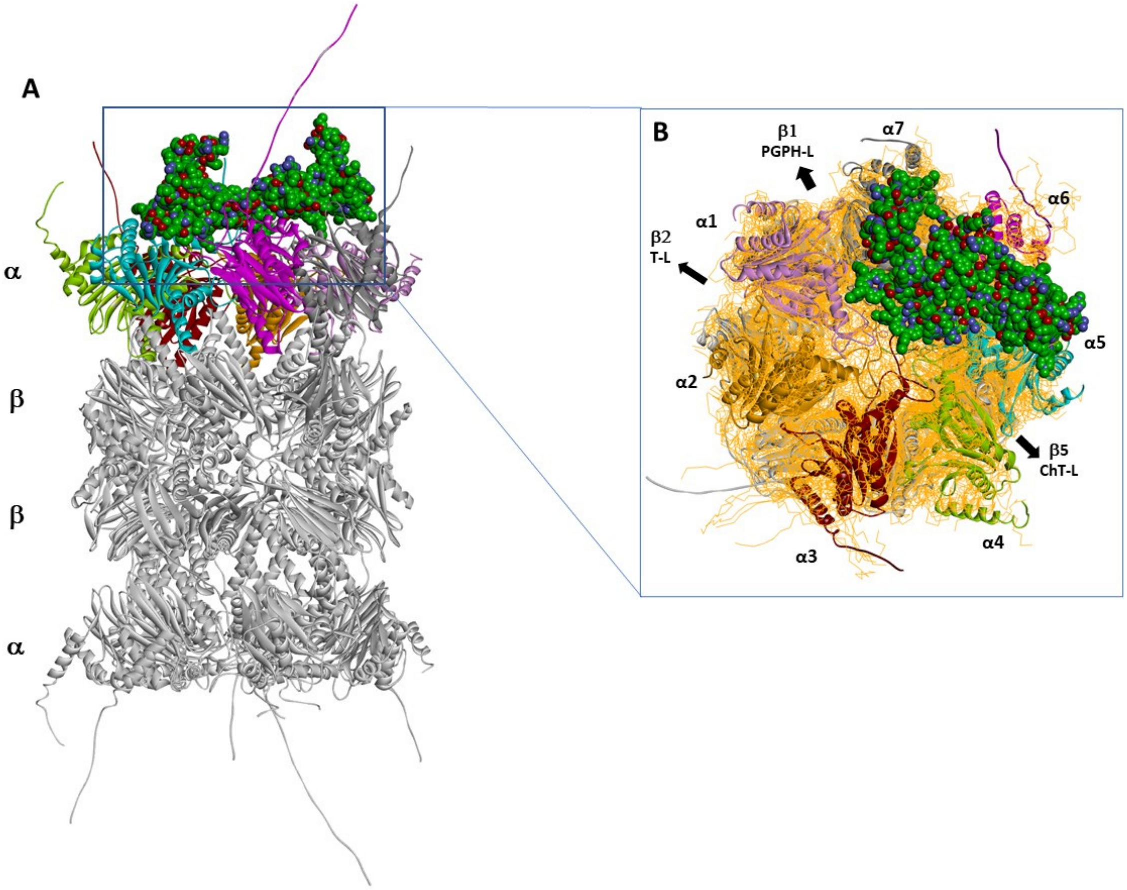

Figure S3. Superimposition by Cα atoms of all the **MTPyApi**/h20S complexes generated by dynamic docking calculations (starting from the extended conformation of the apidaecin) using as starting structure the closed conformation of the human 20S proteasome. A) Longitudinal view; the backbone of the starting complex is displayed as solid ribbons and colored in pink (α1), orange (α2), brown (α3), light green (α4), cyan (α5), magenta (α6), and gray (α7, α subunits of the second ring and all β subunits); **MTPyApi** is displayed as CPK and colored by atom type (C = green); B) Top view of dynamic docking results without the second ring of α and β subunits. The backbone of the calculated complexes is displayed as line ribbons and colored in orange (B and C). The porphyrin ligands are colored by atom type (C: green N: blue; O: red) and displayed as CPK. In B the α subunits and the catalytic β subunits are labeled. In C the catalytic β subunits are labeled and colored in pink (β1), violet (β2), and cyan (β5).

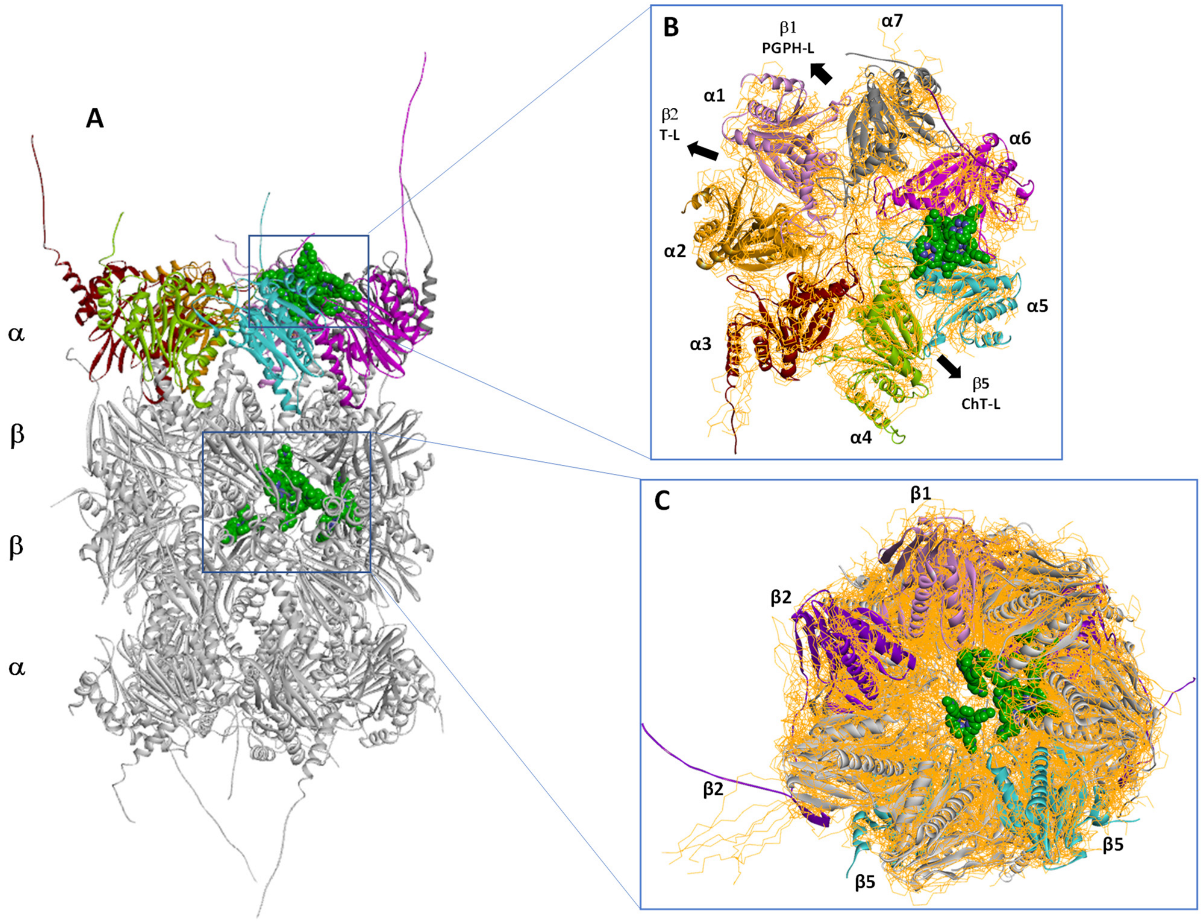

Figure S4. A) Dynamic docking results obtained for **TMPC** using as starting structure one molecule bound at α5-α6 groove of human 20S proteasome in the closed conformation. B) Top view of dynamic docking results without the two rings of β subunits and the second ring of α subunits. C) Top view of dynamic docking results without the first ring of α subunits. The backbone of the starting complex is displayed as solid ribbons and colored in pink (α1), orange (α2), brown (α3), light green (α4), cyan (α5), magenta (α6), and gray (α7, α subunits of the second ring and all β subunits). The backbone of the calculated complexes is displayed as line ribbons and colored in orange (B and C). The porphyrin ligands are colored by atom type (C: green N: blue;) and displayed as CPK. In B the α subunits and the catalytic β subunits are labeled. In C the catalytic β subunits are labeled and colored in pink (β1), violet (β2), and cyan (β5).

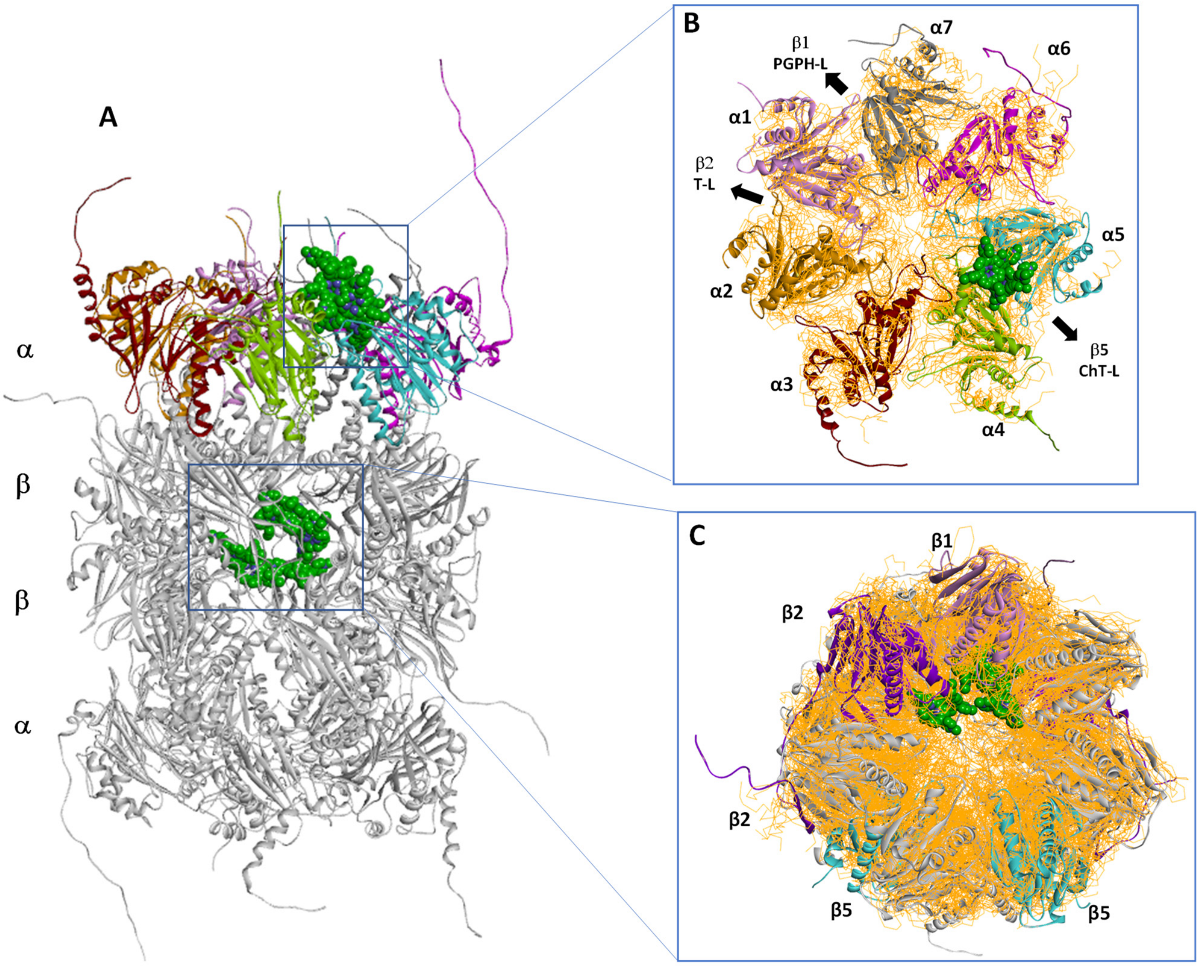

Figure S5. A) Dynamic docking results obtained for **TMPC** using as starting structure one molecule bound at α4-α5 groove of human 20S proteasome in the open conformation. B) Top view of dynamic docking results without the two rings of β subunits and the second ring of α subunits. C) Top view of dynamic docking results without the first ring of α subunits. The backbone of the starting complex is displayed as solid ribbons and colored in pink (α1), orange (α2), brown (α3), light green (α4), cyan (α5), magenta (α6), and gray (α7, α subunits of the second ring and all β subunits). The backbone of the calculated complexes is displayed as line ribbons and colored in orange (B and C). The porphyrin ligands are colored by atom type (C: green N: blue;) and displayed as CPK. In B the α subunits and the catalytic β subunits are labeled. In C the catalytic β subunits are labeled and colored in pink (β1), violet (β2), and cyan (β5).

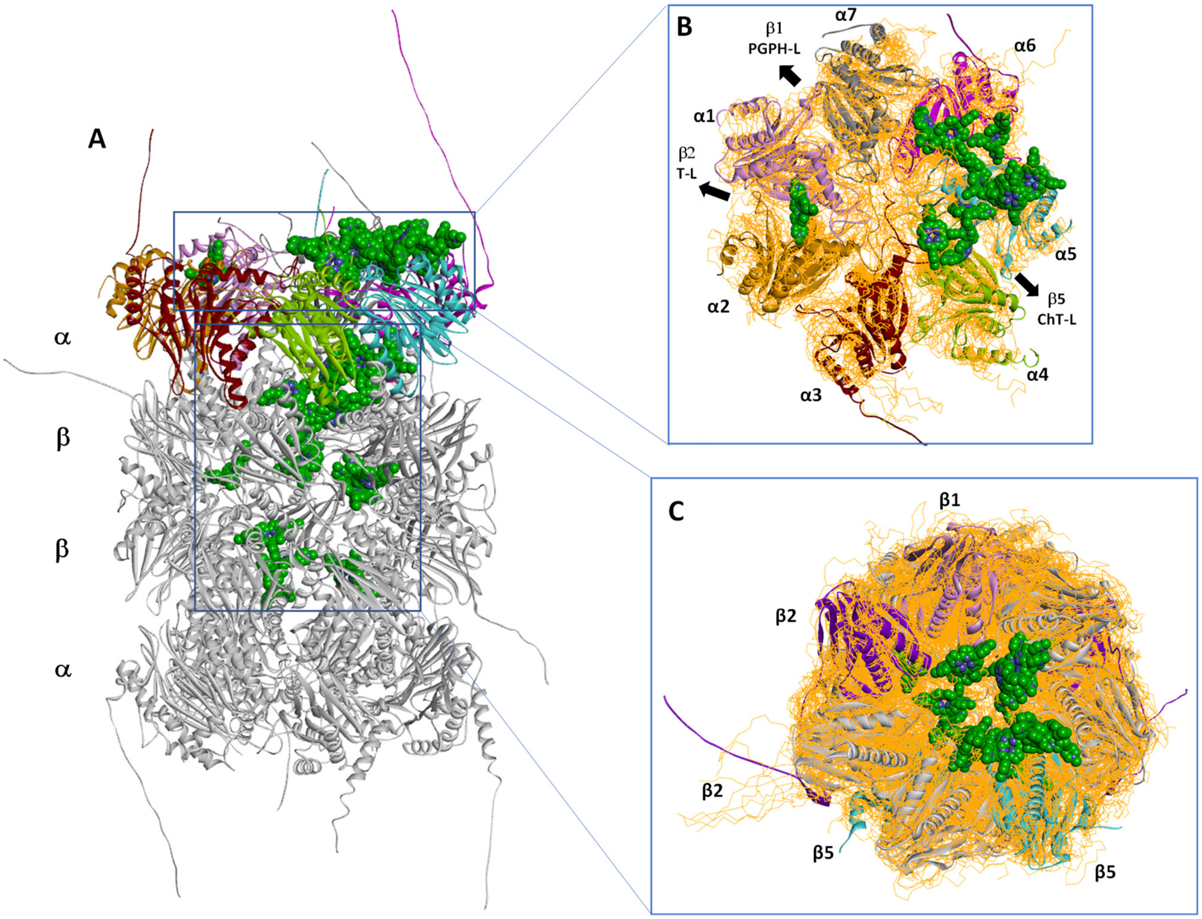

Figure S6. A) Dynamic docking results obtained for **TMPC** using as starting structure three molecules bound at α1-α2, α4-α5 and α5-α6 grooves of human 20S proteasome in the closed conformation. B) Top view of dynamic docking results without the two rings of β subunits and the second ring of α subunits. C) Top view of dynamic docking results without the first ring of α subunits. The backbone of the starting complex is displayed as solid ribbons and colored in pink (α1), orange (α2), brown (α3), light green (α4), cyan (α5), magenta (α6), and gray (α7, α subunits of the second ring and all β subunits). The backbone of the calculated complexes is displayed as line ribbons and colored in orange (B and C). The porphyrin ligands are colored by atom type (C: green N: blue) and displayed as CPK. In B the α subunits and the catalytic β subunits are labeled. In C the catalytic β subunits are labeled and colored in pink (β1), violet (β2), and cyan (β5).

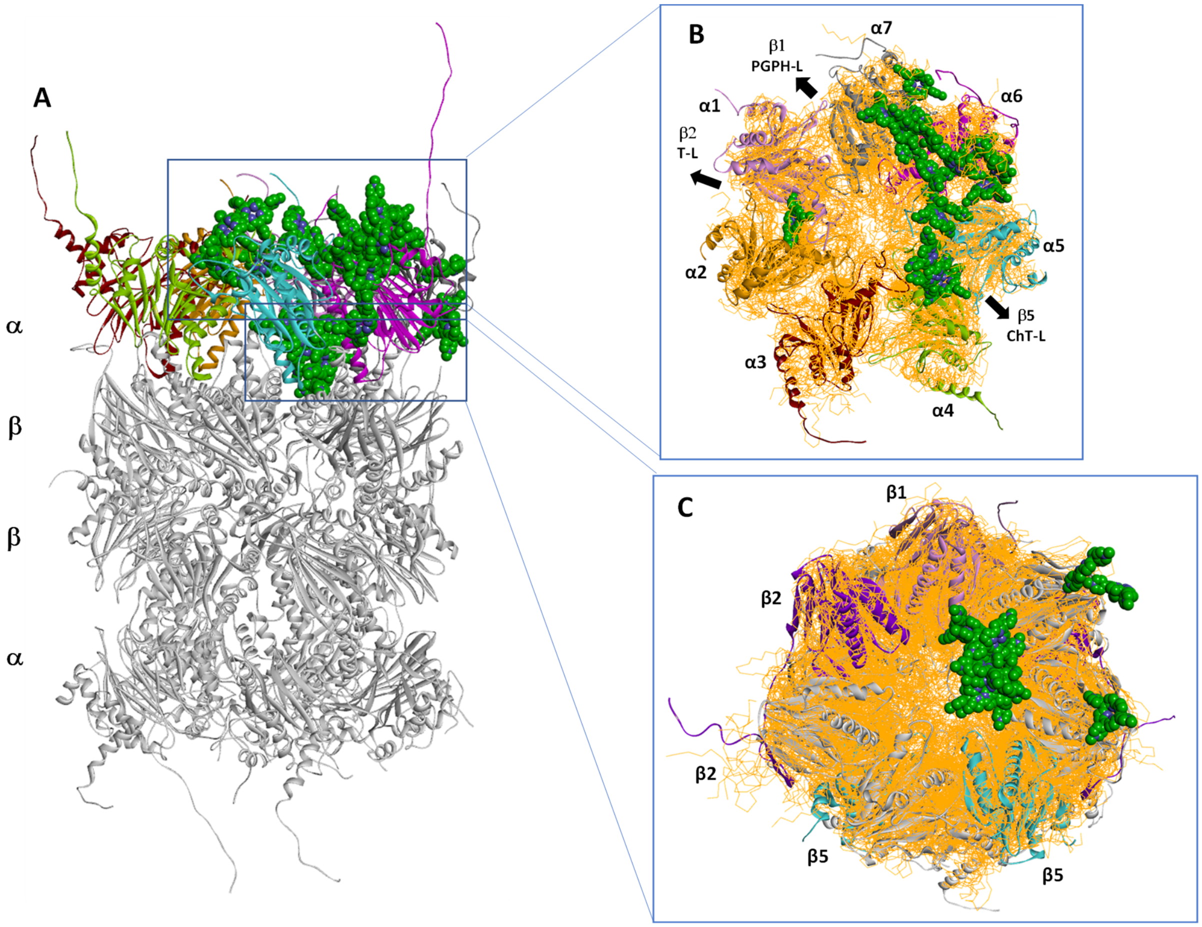

Figure S7. A) Dynamic docking results obtained for **TMPC** using as starting structure three molecules bound at α1-α2, α4-α5 and α5-α6 grooves of human 20S proteasome in the open conformation. B) Top view of dynamic docking results without the two rings of β subunits and the second ring of α subunits. C) Top view of dynamic docking results without the first ring of α subunits. The backbone of the starting complex is displayed as solid ribbons and colored in pink (α1), orange (α2), brown (α3), light green (α4), cyan (α5), magenta (α6), and gray (α7, α subunits of the second ring and all β subunits). The backbone of the calculated complexes is displayed as line ribbons and colored in orange (B and C). The porphyrin ligands are colored by atom type (C: green N: blue) and displayed as CPK. In B the α subunits and the catalytic β subunits are labeled. In C the catalytic β subunits are labeled and colored in pink (β1), violet (β2), and cyan (β5).

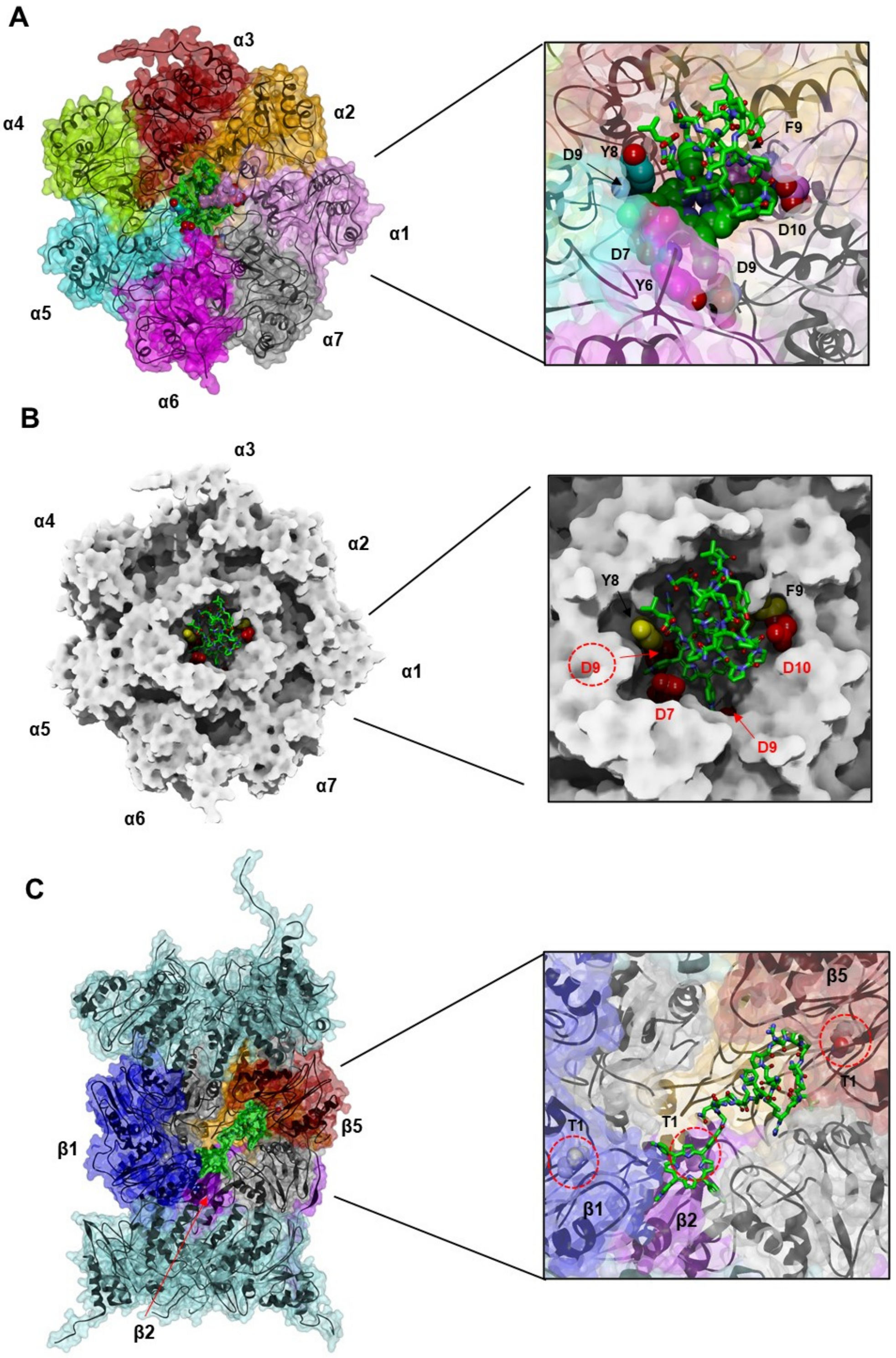

Figure S8. Selected docked complexes of **MTPyApi** bound to human 20S (closed conformation) obtained starting from the folded conformation of the apidaecin. A and B: top and close up view on the ligand binding site at the gate substrate (complex 1). The negative residues interacting with **MTPyApi** and those in common with **H2T4** are displayed in CPK. A) h20S backbone is displayed as black solid ribbons while the transparent Solvent Accessible Surface (SAS) and the displayed residues are colored in pink (*α*1), orange (*α*2), brown (*α*3), light green (*α*4), cyan (*α*5), magenta (*α*6), and gray (*α*7). **MTPyApi** is colored by atom type (C: black; N: blue; O: red) and its SAS is displayed (green). B) h20S SAS is displayed as solid and colored in white. **MTPyApi** is colored by

atom type (C: green; N: blue; O: red) and displayed in stick. The h20S interacting residues are colored: negatively charged=red, aromatic= yellow. The negative residues involved in ionic interactions with RPs are evidenced with a red dashed circle. C) Transversal view and close up view on the ligand binding site at the interface between the first and the second β-ring (complex 7). For clarity of presentation just a cross section of h20S proteasome is shown. h20S backbone is displayed as black solid ribbons while the surface are colored in cyan (α subunits), blue (β1), violet (β2), brown (β5) and orange (β6).

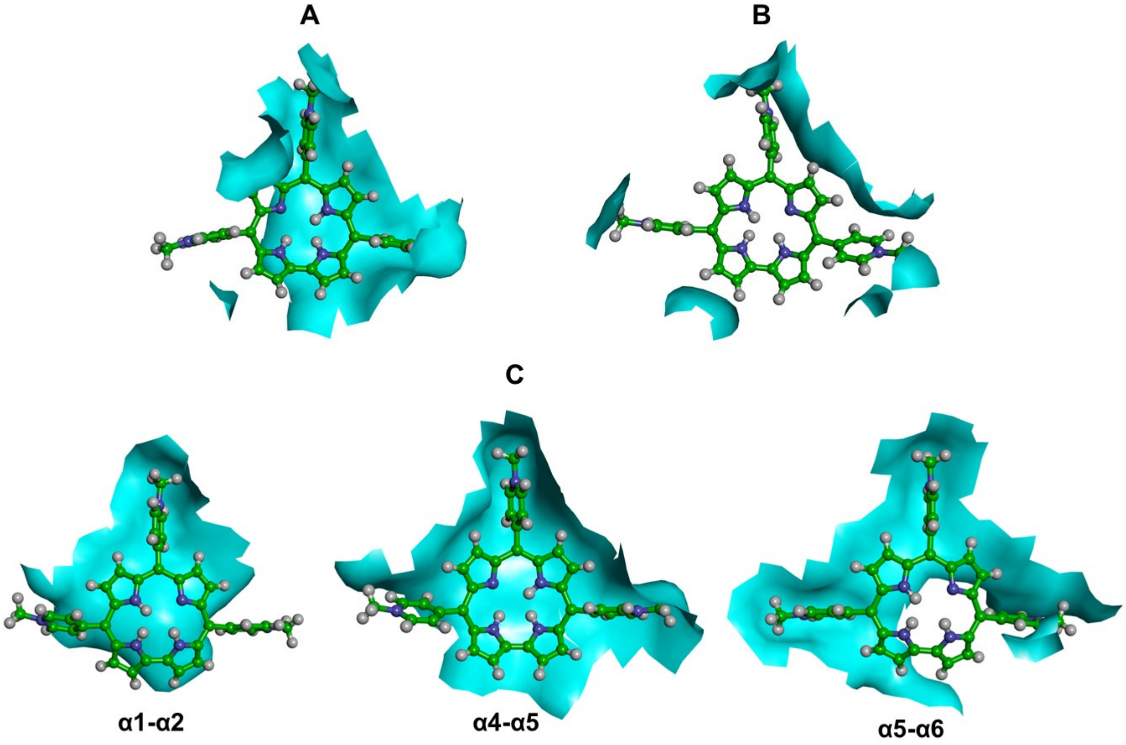

Figure S9. Solvent accessible surface (SAS) of the: one **TMPC** molecule docked to the closed h20S conformation (A), one **TMPC** molecule docked to the open h20S conformation (B) and three **TMPC** molecules docked to the closed h20S conformation (C) (view from the interior to the exterior of the protein). SAS is coloured in cyan and displayed as solid surface; ligand molecules are coloured by atom types (C = green; N = blue; H= white).
